# Lowering Impedance and Improving Sensitivity in Laser-Induced Graphene Biosensors via Speed-Dependent Sequential Irradiation

**DOI:** 10.1021/acsami.5c20377

**Published:** 2025-12-29

**Authors:** Moataz Abdulhafez, Elisa Castagnola, Mirza Sahaluddin, Giulia Baglieri, Golnaz N. Tomaraei, Soumalya Ghosh, Xinyan Tracy Cui, Mostafa Bedewy

**Affiliations:** † Department of Mechanical Engineering and Materials Science, 6614 University of Pittsburgh, 3700 O’Hara Street, Pittsburgh, Pennsylvania 15261, United States; ‡ Department of Industrial Engineering, University of Pittsburgh, 3700 O’Hara Street, Pittsburgh, Pennsylvania 15261, United States; § Department of Bioengineering, University of Pittsburgh, 3700 O’Hara Street, Pittsburgh, Pennsylvania 15261, United States; ∥ Department of Biomedical Engineering, Louisiana Tech University, Ruston, Louisiana 71272, United States; ⊥ Institute for Micromanufacturing, Louisiana Tech University, Ruston, Louisiana 71272, United States; # Department of Neurosurgery, Louisiana State University Health Sciences, Shreveport Louisiana 71103, United States; ∇ Department of Chemical and Petroleum Engineering, University of Pittsburgh, 3700 O’Hara Street, Pittsburgh, Pennsylvania 15261, United States

**Keywords:** laser-induced graphene, finite-element heating, graphitization, impedance, conductivity, neural interfaces, biosensors, flexible devices

## Abstract

Laser-induced graphene (LIG) is a promising material platform for flexible bioelectronic devices, but further lowering its electrochemical impedance, without additives or postprocessing, is essential for scalable, implant-grade microelectrodes. Here, we introduce a speed-dependent sequential irradiation strategy that decouples the kinetically limited carbonization of the first lase from the higher-temperature graphitization induced during the second lase. We show that two passes at 49 mm/s reduce the electrochemical impedance by an order of magnitude compared to a single pass at 105 mm/s. Complementary structural and chemical characterization (Raman spectroscopy, X-ray diffraction, X-ray photoelectron spectroscopy, and transmission electron microscopy) reveals that relasing increases graphitic crystallinity, decreases heteroatom content, and generates a high-surface-area cratered morphology. Finite-element moving-heat-source simulations confirm that the second lasing pass produces markedly higher peak temperatures due to enhanced absorptance and reduced thermal diffusivity of the preformed LINC layer, providing quantitative thermodynamic support for the observed graphitization and controlled ablation. These combined effects enable robust, low-impedance microelectrodes capable of detecting dopamine concentrations below 25 nM using square-wave voltammetry. Importantly, this performance is not achievable using single-pass LIG. We further demonstrate seamless spatial control of the morphology on the same substrate, underscoring the versatility of the approach. Sequential CO_2_-laser irradiation therefore offers a scalable, additive-free pathway toward high-performance carbon microelectrode arrays for next-generation neural sensing and stimulation technologies.

## Graphical abstract

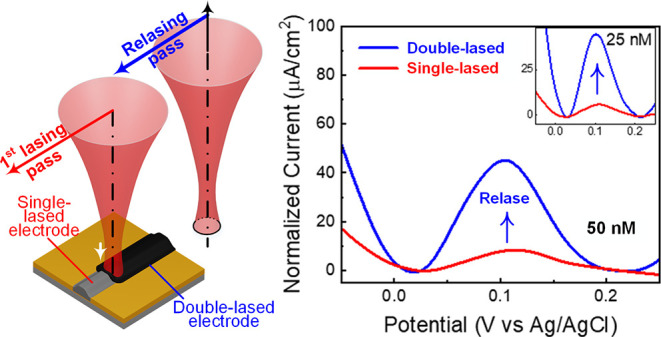

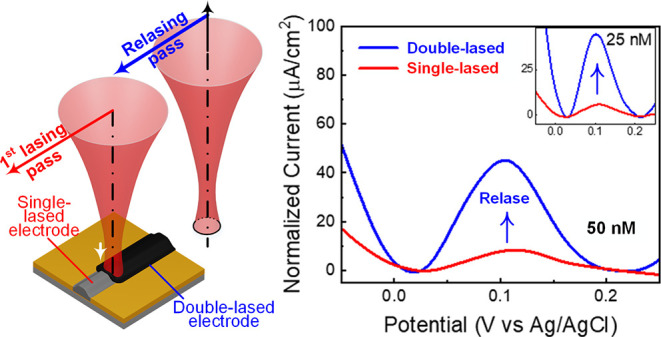

## Introduction

Laser direct writing of graphene on polymers is an attractive technique for the fabrication of functional electrodes and interconnects on flexible devices.^1–3^ In this process, laser irradiation of the solid polymeric precursor leads to thermally driven carbonization, which creates porous graphene patterns as the laser is scanned over the substrate.^4^ The produced three-dimensional graphene material is referred to as laser-induced graphene (LIG),^4^ laser-engraved graphene (LEG),^3^ or laser-induced nanocarbon (LINC).^5^ In this work, we will use the term LINC to collectively refer to all types of nanocarbon morphologies that are generated by this process, including isotropic porous structures, anisotropic cellular networks, and woolly fibrous morphologies.^5–7^ From a manufacturing perspective, LINC offers multiple key advantages: (1) direct-write nature, which enables facile patterning of any desired device design; (2) inherently fast and cheap, which facilitates scalability owing to its roll-to-roll compatibility; (3) no need for transfer or printing of graphene, such as by inkjet printing,^8–10^ on a flexible substrate, which eliminates the need for creating graphene-loaded inks.^11–14^ Accordingly, the flexibility of the LINC process, in addition to the functionality of the fabricated graphene, enables a wide range of applications like heaters for efficient boiling applications,^15^ wearable electronics,^3^ sensors,^16^ microfluidics,^17–19^ drop manipulation,^20^ battery applications,^21^ and actuators.^22^ Additionally, the porous nature of LINC structures makes them ideal for electrochemical biosensors.^18,23–25^ For example, LINC-based sensors have been used for detection of biomolecules^3^ like dopamine,^24–27^ uric acid,^18,27^ and interleukin.^28^ Nevertheless, further reduction of the impedance of LINC electrodes is the key toward increasing the detection sensitivity down to the nanomolar range.

Previous efforts toward enhancing the electrical conductivity of LINC have generally focused on increasing laser power and lowering speed,^29,30^ multiple passes,^31^ and beam spot overlap.^32^ In addition to implementing heuristics, statistical design of experiments and machine learning tools have also been employed.^33,34^ Nevertheless, many of these approaches focus on larger LINC areas in the millimeter-to-centimeter range^16^ and use sheet resistance in Ω/□ as a measure of resistance,^4,29^ which assumes a uniform thin film of LINC and is not practical for eventual use in microelectrode applications, wherein individual lines need to be in the micrometer range. Hence, we focus here on fabricating individual LINC electrodes with a line width that is determined by the beam spot size without any spot overlap or rastering. Importantly, single-line lasing in vector mode enables development of a fundamental mechanistic understanding of the effects of different laser parameters (e.g., speed and power) on surface morphology without the influence of laser pass overlap or proximity effects among high-density lines or pixels in the typical rastering mode. Accordingly, our work aims to create functional LINC electrodes of high-performance biosensors with reduced impedance and tunable morphology, which solves reported issues with LINC-based single-line electrodes.^27^

For in vivo electrochemical detection of neurotransmitters, the gold-standard electrode is made of a single carbon fiber.^35–39^ To achieve high-resolution multisite detection, arrays of carbon fibers have been made, but the fabrication process is manual and lacks reproducibility and scalability.^40–42^ By including pyrolysis in the lithographic fabrication process, glassy carbon microelectrode arrays (MEAs) with planar^43–45^ or fiber-like^46^ configurations with metal interconnects have been batch-produced and are capable of multisite dopamine and serotonin sensing in the brain. Alternatively, MEAs with GC electrodes and GC interconnects on thin, flexible substrates have also been successfully fabricated using a double pattern-transfer photolithographic process, including transfer-bonding on temporary polymeric support.^47,48^ The limitations with these lithographically made GC MEAs are: (1) the electrode is glassy carbon, which has low surface area and conductivity, and (2) the fabrication process requires the use of a clean room facility with complicated processing steps. The possibility of using laser-writing techniques to pattern nanocarbon electrodes and interconnections on a flexible polymeric substrate would greatly simplify the fabrication process of carbon-based MEAs, reducing the production time and cost.

However, for LINC to be used in this application, we need to achieve both a high-resolution process and high-conductivity carbon materials with tailored properties, such as porosity and surface area, without introducing any additional components, such as metal nanoparticles, that present major toxicity concerns. Indeed, prior work on enhancing LINC sensing performance and improving electrode conductivity relied on integrating additive nanostructures like metal nanoparticles^49,50^ or conductive polymers like PEDOT.^26^ In contrast, in this work, we lower electrode impedance and increase conductivity by controlling the kinetics of lasing in each of two consecutive passes.

Our approach of sequential irradiation at different speeds offers two main advantages: (1) It allows the decoupling of the polyimide carbonization process in the first lasing step from the graphitization in the second step, enabling precise control over both morphology and crystallinity; (2) the unique combination of high electrical conductivity and high surface area of our porous graphene results in low electrochemical impedance, significantly enhancing the sensitivity of basal dopamine (DA) detection at concentrations below 25 nM, using a previously optimized square wave voltammetry (SWV) technique.^38^ Additionally, the scalability of the LINC process and its tunable properties make it a promising technology for next-generation implantable microelectrode arrays and other neural interface applications.

## Results and Discussion

### Relasing for Controlling Surface Topography and Morphology of LINC Electrodes

In a typical LINC line fabrication process, a laser beam with a power *P* lases a polyimide sheet with a speed *v*_1_. The sample surface is at a distance *z* from the beam waist (assumed Gaussian), which decides beam spot size 2*w*. A schematic of the experimental setup is shown in Figure 1a. A continuous CO_2_ laser (λ = 10.6 μm) is used for this study, which has been characterized to estimate its beam parameters in previous work. The laser profile is shown in SI Figure S1. Upon beam exposure, the polyimide absorbs the laser at the wavelength λ = 10.6 μm strongly.^51^ This is followed by a temperature increase in the material, which thermally drives the carbonization of the polyimide. After this first lasing pass, a second lasing process is followed with speed *v*_2_ at the same power *P* as presented in Figure 1b until it is fully relased. In what follows, “single-lase” refers to electrodes fabricated using a single laser pass, whereas “double-lase” or “relased” refers to electrodes that undergo a second laser pass (relasing). To illustrate the effects of relasing, the second lasing process is halted midway during the process and imaged using SEM at the interface, Figure 1c. These lines are lased using laser conditions *P* = 12.5 W and different *v*_1_ ranging between 269 and 105 mm/s and a fixed *v*_2_ = 49 mm/s. The SEM images showing this transition (before and after relasing) are presented in Figure 1c and SI Figure S2. Figure 1c demonstrates the gradual change of morphology at the interface between LINC lines resulting from a single lase and the relased portion. The effects of the relasing step include evidence of ablation of the porous surface of the LINC lines^52^ and an increase in the surface porous carbon volume and width of the LINC lines. The single LINC lines resulting from a single lase at *v*_1_ = 269 mm/s (before relasing) exhibit a porous surface morphology, as shown in the SEM image Figure 1e. After relasing, SEMs reveal a higher surface area cellular network, as shown in Figure 1f.

**Figure 1:**
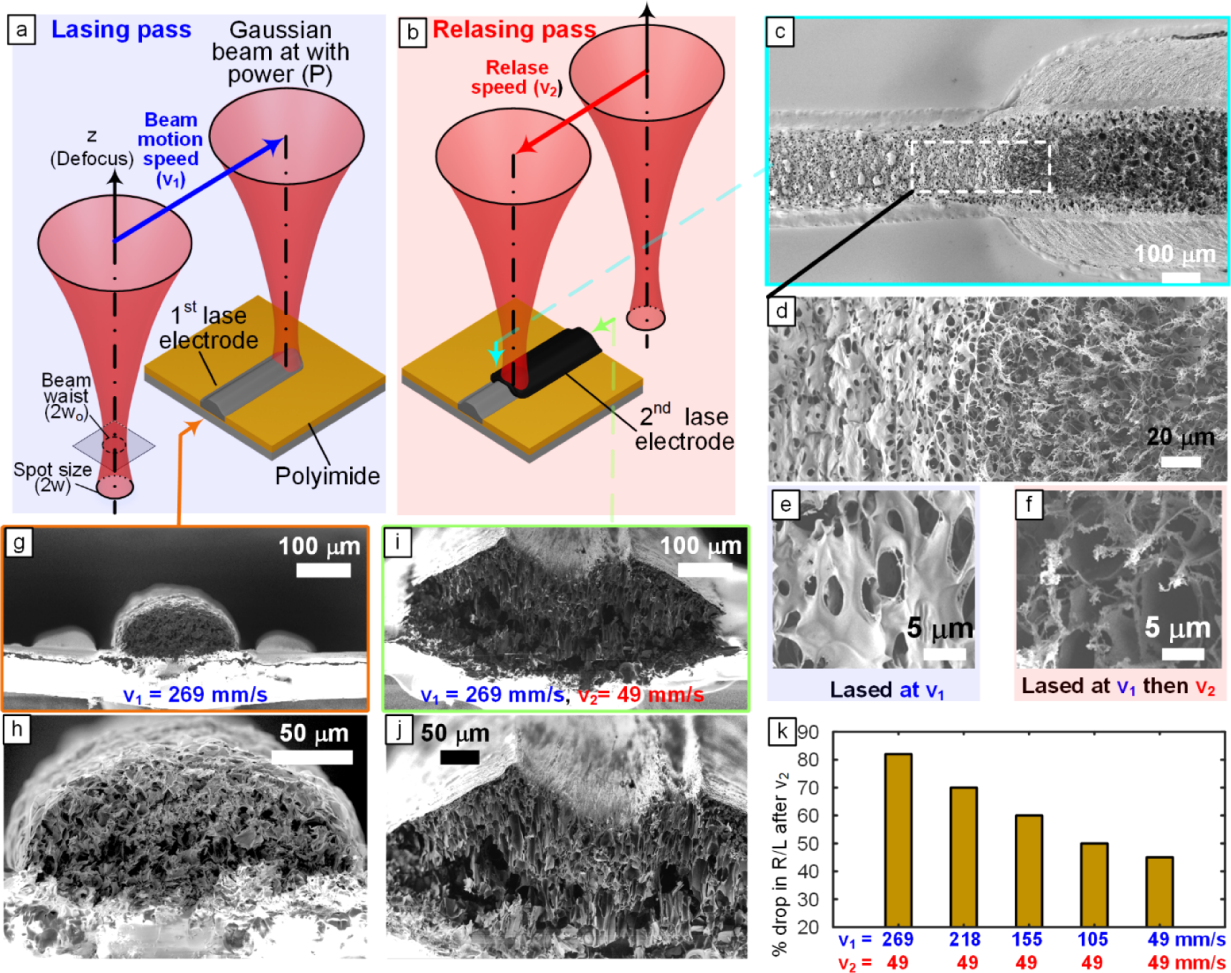
Schematic illustrating the first lasing step (single-lase) at speed *v*_1_ (a) and the second lasing step (relase) at speed *v*_2_ (b). (c) SEM image of the interface between the line lased at *P* = 12.5 W, *z* = 9 mm, and *v*_1_ = 269 mm/s and the remaining line section relased at *v*_2_ = 49 mm/s. (d) SEM image illustrating the influence of the relasing on surface morphology with higher resolution imaging in panels (e) and (f). (g,h) SEM images of the cross section of single-lased LINC lines at *v*_1_ = 269 mm/s. (i,j) SEM images of the cross section of relased LINC lines at *v*_1_ = 269 mm/s and relase speed *v*_2_ = 49 mm/s. (k) Bar chart illustrating the influence of relasing on electrical resistance reduction (% drop in *R*/*L* after *v*_2_).

SI Figure S2a–d shows the influence of the second lasing step on LINC lines lased at slower speeds, in which the surface morphology transitions from a porous surface morphology into a cellular morphology and an ablated morphology at even lower speeds. The relasing pass generally results in ablation on the top layer of the LINC lines and an increase in the width of the lines, an effect that decreases as *v*_1_ approaches *v*_2_. It is also clear from the SEM images that the extent of ablation in the red lines is more extensive at lower *v*_1_ speeds. Cross-sectional SEM imaging of the LINC lines reveals the inner morphology of the LINC lines and the influence of relasing. SEM imaging of the cross sections of LINC lines lased at *v*_1_ = 269 mm/s and another line lased at the same *v*_1_ and then *v*_2_ = 49 mm/s are shown in Figure 1g,h and Figure 1ij, respectively. These images show the increase in cross-section and change in the shape of the LINC lines after relasing. The lines obtained at a high-speed first lasing step (269 mm/s) are originally dome-shaped with a porous outer shell; upon relasing at low speed (49 mm/s), the line width increases, and an ablation crater is revealed on top of the line, as well as some change to the inner morphology. High-resolution SEM imaging of the cross sections of mechanically fractured LINC lines is also imaged to reveal more detail of their inner morphology in SI Figure S3a,b. Electron micrographs reveal the highly desirable porous morphology, wherein the volume of the cross sections of LINC lines is mostly empty and composed of cells/pores with very thin walls, indicating a high-surface-area morphology, which is ideal for electrochemical sensing. Some difference in the inner morphology is noted between the cross section of the lines lased at *v*_1_ = 269 mm/s and the lines obtained at *v*_1_ = 49 mm/s and *v*_2_ = 49 mm/s. In the first case, the cell features are not clearly directional and feature more wrinkles and a porous outer wall structure with fibrous features underneath. On the other hand, the relased case reveals anisotropic features, with a lot of the cell walls having a vertical alignment and an ablated top showing evidence of material removal (cratering). Additional high-resolution SEM imaging in SI Figure S4 reveals that the thickness of the cell walls ranges between 20 and 50 nm. In addition to the surface morphological changes due to relasing, there is also a clear resistance-per-length drop due to the relasing step, illustrated in Figure 1k, with resistance-per-length drops going from a value of 80% at *v*_1_ = 269 mm/s and *v*_2_ = 49 mm/s to 40% at *v*_1_ = 49 mm/s and *v*_2_ = 49 mm/s.

### Relasing Speed Is Key for Lowering LINC Electrical Resistivity

While relasing generally reduces the electrical resistance of LINC electrodes, it is important to determine whether relasing reduces the effective electrical resistivity of the material or just increases the cross-section. Another important question is, how low can the effective resistivity be without excessively damaging or cutting the electrodes? To answer these questions, another study with fixed *v*_1_ and changing *v*_2_ is carried out. To select *v*_1_, a single-pass speed study is completed first, with the results shown in Figure 2a,b. Figure 2a demonstrates the effect of the lasing speed on the resistance per electrode length at fixed power *P* = 12.5 W and *z* = 9 mm. The onset of damage from excessive electrode fracture and cutting is illustrated in the figure as a shaded region at excessively low speeds. The resistance per length (*R*/*L*) drops from a value of 43 Ω/mm at *v*_1_ = 105 mm/s to a value of around 14 Ω/mm at *v*_1_ = 49 mm/s. The trend shows that resistance per length can potentially be lower; however, the line would eventually be excessively damaged due to fracture or cutting. By measuring the cross-sectional areas of the electrodes using SEM imaging, the effective resistivity of the electrodes can be calculated. This “effective resistivity” is a geometry-based, effective metric derived from *R*/*L* and the measured cross-sectional area of the electrodes, rather than an absolute bulk resistivity, while still providing a practical way to compare changes in electrode resistivity between single-lase and relase conditions. The corresponding effective resistivity values to *R*/*L* results in Figure 2a are shown in Figure 2b. A similar trend in electrical resistivity reduction with slower lasing speeds is observed, but with a less steep rate of decrease at lower *v*_1_. The value of the effective resistivity ranges between 0.47 Ω·cm at *v*_1_ = 155 mm/s and 0.14 Ω·cm at *v*_1_ = 49 mm/s, beyond which the electrodes are excessively damaged. To investigate the effect of relasing, the first lasing speed is fixed at *v*_1_ = 49 mm/s, while *v*_2_ is changed between 155 and 29 mm/s. *R*/*L* values are shown for *P* = 12.5 W and *P* = 18.3 W with *v*_1_ = 49 mm/s in Figure 2c. The corresponding effective resistivity values at *P* = 12.5 W are shown in Figure 2d. The *R*/*L* values range from 13 Ω/mm to 5 Ω/mm for *v*_2_ values between 155 and 29 mm/s, respectively, with a decreasing trend. The corresponding effective resistivity values are noted to drop from around 0.14 Ω·cm to 0.08 Ω·cm. The relasing does not seem to affect the resistivity at high *v*_2_ = 155 mm/s but has a stronger decreasing effect as *v*_2_ approaches *v*_1_, highlighting the importance of a lower-speed relase for achieving a significant reduction of LINC resistivity. However, as *v*_2_ is decreased below *v*_1_, excessive damage is noted in the electrodes.

**Figure 2:**
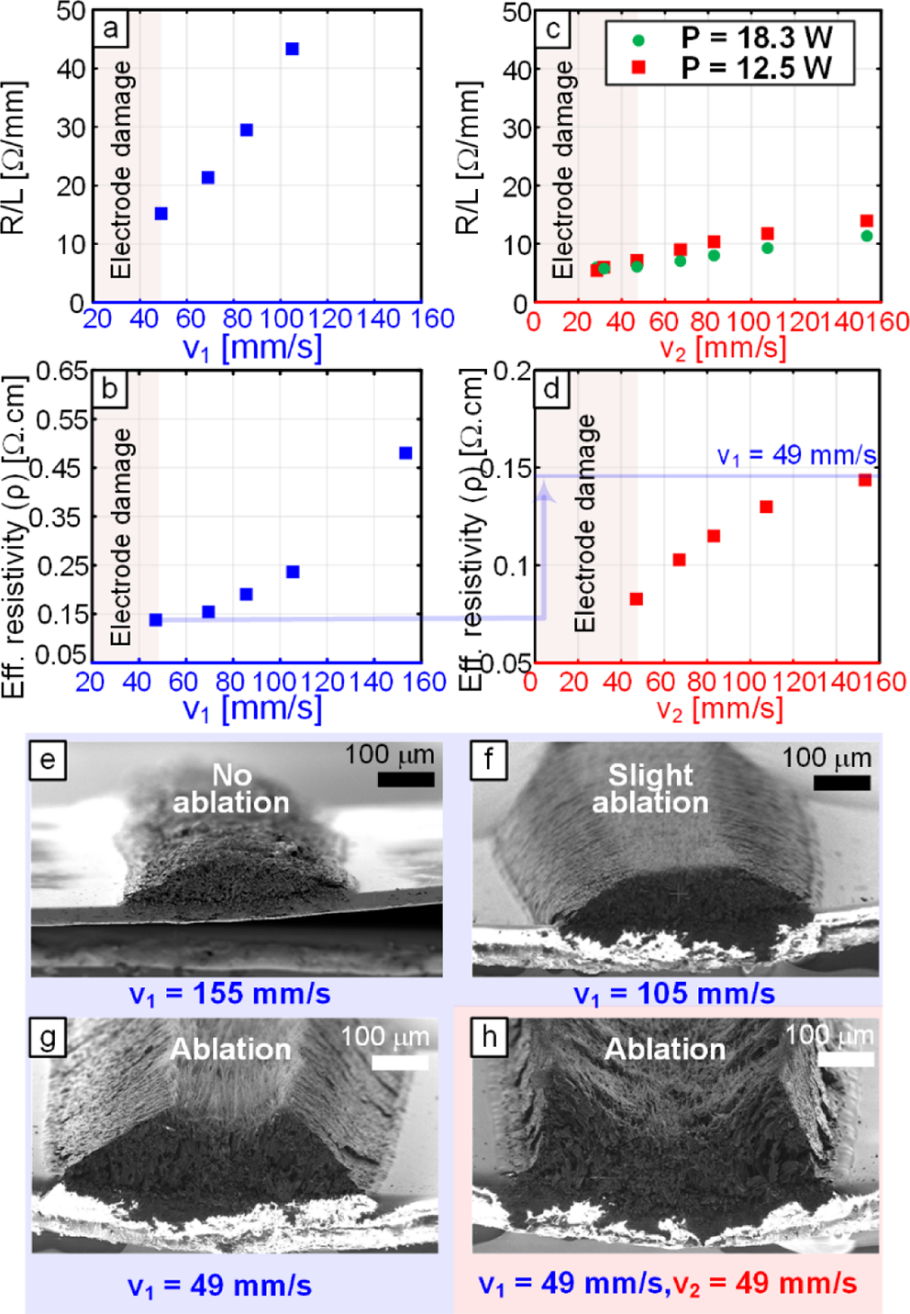
(a) Plot illustrating the influence of lasing speed on resistance per length values for LINC electrodes at *P* = 12.5 W and *z* = 9 mm. The onset of electrode damage at low speeds is illustrated. (b) Plot of corresponding effective resistivity of LINC electrodes in (a). (c) Plot illustrating the influence of relasing speed on resistance per length values for LINC electrodes at *P* = 12.5 W, *z* = 9 mm, *v*_1_ = 49 mm/s and *P* = 18.3 W, *z* = 9 mm, *v*_1_ = 49 mm/s. (d) Plot of corresponding effective resistivity of LINC electrodes in (c). (e,f,g) SEM images illustrating the cross-sections of LINC electrodes at *P* = 12.5 W, *z* = 9 mm, and different lasing speeds. (h) SEM image illustrating the cross-sections of LINC electrodes at *P* = 12.5 W, *z* = 9 mm, and *v*_1_ = 49 mm/s and *v*_2_ = 49 mm/s.

To further verify that the observed improvements originate from intrinsic changes in the LINC material rather than geometric or measurement artifacts, we performed an additional set of electrical measurements on single-lased and relased electrodes, complementing the results in Figure 2. For a fixed degree of defocus (*z* = 9 mm) and scan speed (111 mm·s^–1^), the resistance per unit length of individual electrode lines was measured in air and plotted as a function of laser power (SI Figure S5a,b). Across the examined power range, relased electrodes consistently exhibit a substantially lower *R*/*L* than single-lased electrodes, with small standard errors over *n* = 3 samples, confirming that the conductivity enhancement is robust and reproducible. To confirm that this relasing effect extends beyond individual line electrodes, we further fabricated LINC areas and measured their sheet resistance using a four-point probe in a van der Pauw configuration for single-lased and relased conditions fabricated at *P* = 12.5 W, *v* = 111 mm·s^–1^, *z* = 6 mm, and a raster gap of 355 μm. The relased areas show a marked decrease in sheet resistance compared to single-lased areas (SI Figure S5c), in agreement with the line resistance trends.

Taken together, our results clearly demonstrate that relasing is an effective technique to reduce the resistivity of LINC electrodes beyond the limits of single-pass lasing with speed-dependent tenability of morphology and properties. In fact, the effective resistivity exhibits a strong speed dependence, which we will further investigate through other characterization techniques below. SEM imaging of LINC electrode cross-sections is shown in Figure 2e–h to demonstrate the effect of lasing and relasing speed on the shape of the cross-section and to show the extent of top surface ablation. Figure 2e,f shows the transition from wrinkled and porous surface morphology with a small cross-sectional area to a more dome-shaped cross-section with some indications of ablation (material removal) on the top of the dome when *v*_1_ decreases from 155 to 105 mm/s. Figure 2g shows the influence of further decreasing the *v*_1_ to 49 mm/s: the ablation of the top dome of the electrodes, revealing some of the internal cells of the material. Figure 2h clearly shows the effect of relasing on the cross-section, with much more top surface ablation creating a U-shaped crater, exposing even higher surface area with porous internal morphology. This effect can be exploited to control the mesoscale surface morphology of LINC, as illustrated in SI Figure S6. With decreased relasing speed, more of the top surface is ablated, allowing for control over porosity of LINC, which is quantified using image processing. We show that with decreasing *v*_2_ from 155 to 28 mm/s, the solid area fraction is reduced from about 66% to about 54%.

### Influence of Relase Speed on Electrode Graphitic Crystallinity and Surface Chemistry

To understand the driving factors behind the decrease in the electrical resistivity of LINC electrodes and its speed dependence, we characterize the effect of relasing on the crystallinity and surface chemistry of LINC. We use Raman spectroscopy and X-ray photoelectron spectroscopy (XPS). Additionally, to understand the changes in the inner LINC morphology, X-ray diffraction and transmission electron microscopy (TEM) are performed.

Initially, Raman spectra of lines generated using different lasing speeds *v*_1_ are analyzed. The influence of relasing is then investigated by fixing *v*_1_ at 49 mm/s and changing the *v*_2_ between 155 and 28 mm/s at a fixed power *P* = 12.5 W and defocus level *z* = 9 mm. The results of the Raman analysis are shown Figures 3a–d and SI S7. The Raman spectra for the lasing study are shown in Figure 3c, and the Raman spectra for the relasing study are shown in Figure 3d. The speed dependence of the *I*(D)/*I*(G) ratio for both studies is plotted in Figure 3a,b. The *I*(D)/*I*(G) ratio and peak sharpness are generally highly correlated to the quality of sp^2^ carbon and the graphitic crystallite size. Specifically, a smaller *I*(D)/*I*(G) ratio indicates increasing crystallite size according to the Tuinstra–Koenig correlation and is also related to defect levels in the material.^53^ In both studies, the *I*(D)/*I*(G) is shown to decrease with decreased speed, owing to the higher laser fluence delivered at lower speeds. For a LINC line lased at a speed of *v*_1_ = 105 mm/s, the *I*(D)/*I*(G) ratio is 0.65, and it drops to 0.44 at *v*_1_ = 49 mm/s, as shown in Figure 3a,c. Importantly, relasing demonstrates that the value can be reduced further to an average ratio of around 0.26 (which can be as low as 0.1) at a relasing speed of *v*_2_ = 31 mm/s, as shown in Figure 3b,d. Furthermore, it is observed that the *I*(2D)/*I*(G) ratio also decreases with decreasing speed, as shown in SI Figure S7. The *I*(2D)/*I*(G) ratio is typically associated with graphitic layer stacking. These results suggest that the observed speed dependence of the resistivity is correlated to increased graphitization, promoting the size and crystallinity of the sp^2^ domains. This graphitization is driven by the increased dwell time of the laser beam when speeds are decreased. These conclusions are also supported by XRD analysis.

**Figure 3:**
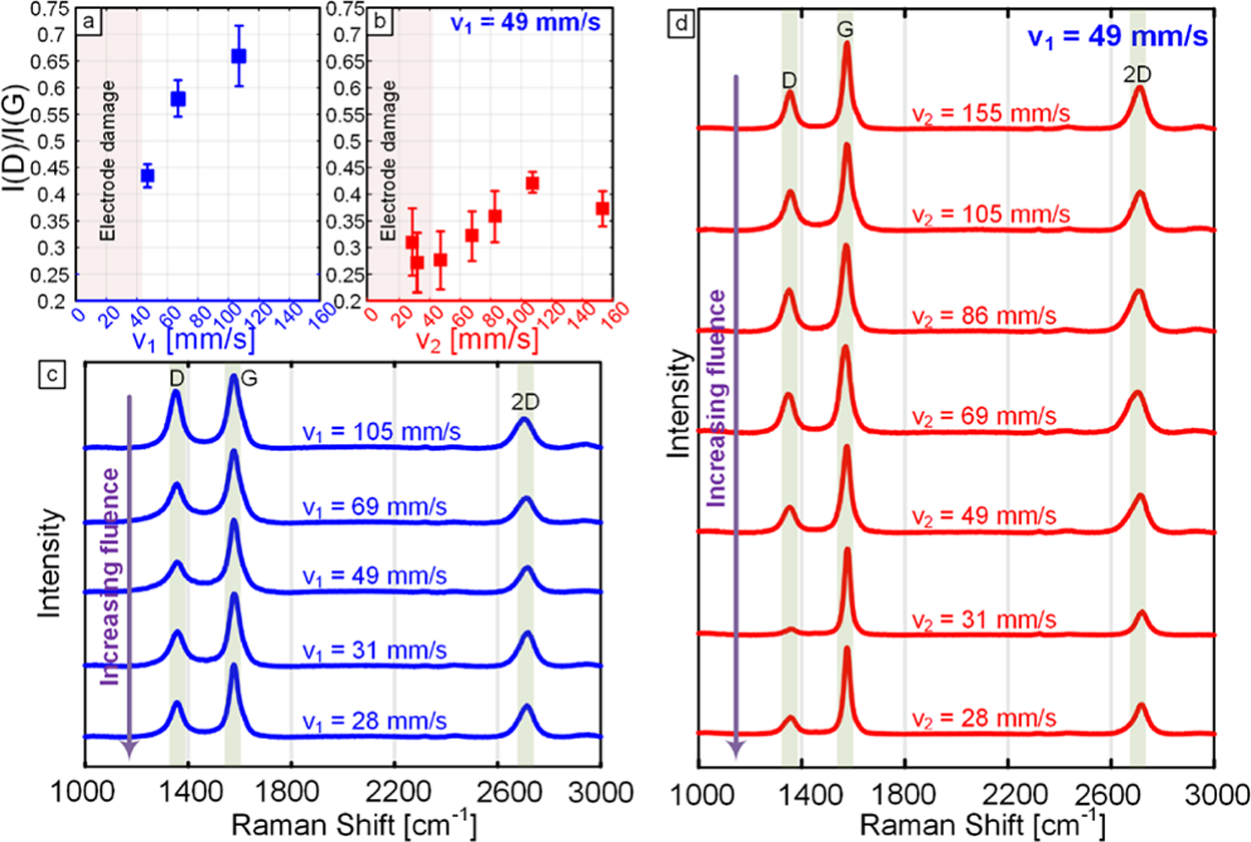
(a) Plot illustrating the influence of lasing speed on the *I*(D) to *I*(G) ratio for LINC lines lased at *P* = 12.5 W, *z* = 9 mm, with each point representing the mean of 5 measurements and error bars representing sample standard error. (b) Plot illustrating the influence of lasing speed on the *I*(D) to *I*(G) ratio at *P* = 12.5 W, *z* = 9 mm, and *v*_1_ = 49 mm/s, with each point representing the mean of 5 measurements and error bars representing sample standard error. (c) Representative Raman spectra illustrating the effects of lasing speed at *P* = 12.5 W, *z* = 9 mm. (d) Representative Raman spectra illustrating the effects of relasing speed at *P* = 12.5 W, *z* = 9 mm, and *v*_1_ = 49 mm/s.

XRD profiles of LINC from lines lased at *v*_1_ = 105 mm/s, *v*_1_ = 49 mm/s, and lines lased at *v*_1_ = 49 mm/s and relased at *v*_2_ = 105 mm/s and *v*_2_ = 49 mm/s are shown in Figure 4a. Using the XRD profiles, the graphitic crystallite size along the thickness, as well as the interlayer spacing and number of layers, are analyzed and derived. The analysis is presented in Figure 4b,c, SI Figure S8, and SI Figure S9. The analysis is performed by the deconvolution of the (002) peaks into Lorentzian peaks and using the main peak locations and peak FWHM to derive the crystallite parameters, as illustrated in SI Figure S8. The peak parameters are presented in Table S1 and are plotted in Figure 4 b,c and SI Figure S9. The (002) peaks are generally observed to be asymmetric at the lowest lasing speed (*v*_1_ = 105 mm/s), where the data are noted to have a high noise-to-signal ratio and a weak (100) peak. With decreasing speed and relasing, the asymmetry, represented in the peak shoulder, significantly decreases, with an improved signal-to-noise ratio. The analysis shows that lines lased at *v*_1_ = 105 mm/s have an average *L*_c_ size of around 3 nm with an interlayer distance *d*_002_ of 3.418 Å and 9 graphitic layers. This changes to an average *L*_c_ = 5 nm with *d*_002_ = 3.418 Å and 15 layers when the lasing speed is reduced to *v*_1_ = 49 mm/s. For relased lines at *v*_1_ = 49 mm/s and *v*_2_ = 105 mm/s, *L*_c_ increases to 5.5 nm and *d*_002_ decreases to 3.402 Å with 16 layers. At an even lower relase speed of *v*_2_ = 49 mm/s, more significant changes are noted as the average *L*_c_ increases to 6.5 nm with *d*_002_ = 3.407 Å and 19 layers. The significant decrease in *d*_002_ with relasing suggests a significant reduction in the heteroatom content, as corroborated by the XPS results below. Thus, the XRD analysis not only confirms increased graphitic crystallite thickness and improved stacking but also indicates a progressive transition toward a more graphitic, less heteroatom-rich structure under relasing conditions.

**Figure 4:**
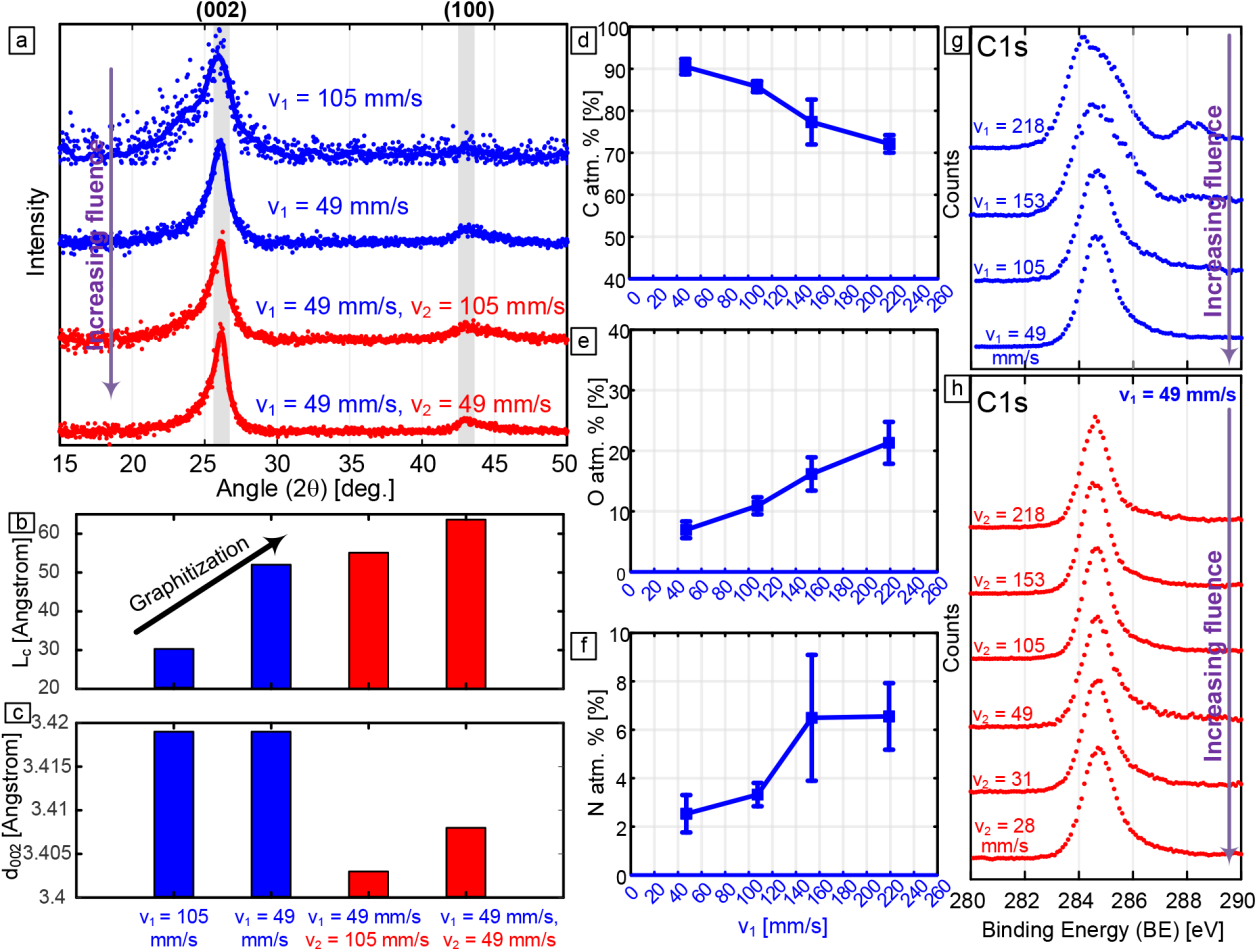
(a) XRD profiles of scrapped LINC lines at *P* = 12.5 W and *z* = 9 mm at different lasing and relasing conditions. (b) Bar chart illustrating the effect of lasing and relasing conditions on *L*_c_. (c) Bar chart illustrating the effect of lasing and relasing condition on *d*_002_. (d) Plot showing the change in percentage atomic content of carbon derived from the XPS survey spectrum of the surface of LINC lines lased at *P* = 12.5 W and *z* = 9 mm, with each point representing the mean of 5 measurements and error bars representing sample standard error. (e) Plot showing the change in percentage atomic content of oxygen derived from the XPS survey spectrum at different lasing speeds. (f) Plot showing the change in percentage atomic content of nitrogen derived from the XPS survey spectrum at different lasing speeds. (g) Plots showing C 1s core scan spectra at different speeds. (h) Plots showing C 1s core scan spectra at different relasing speeds at *P* = 12.5 W, *z* = 9 mm, and *v*_1_ = 49 mm/s.

The results of the XPS analysis of LINC surfaces are shown in Figure 4d–h and SI Figures S10 and S11. The elemental composition (carbon, oxygen, and nitrogen content) for the single-lase study derived is extracted from the XPS survey scans and is shown at different lasing speeds *v*_1_ in Figure 4d–f. With decreasing lasing speed, the heteroatom content (oxygen and nitrogen) is generally observed to decrease, resulting in a significant increase in carbon content. At *v*_1_ = 218 mm/s, the atomic percentage of carbon is around 73%, which increases to a value of 92% at a *v*_1_ speed of 49 mm/s. C 1s core scans are presented in Figure 4g,h, showing the evolution of the shape of the C 1s peaks with changing lasing speed *v*_1_ and relasing speed *v*_2_ (at constant *v*_2_ = 49 mm/s). The deconvolution of C 1s peaks into its different components is presented in SI Figure S11 and SI Table S2. Results show that with relasing, the C=C peak indicative of sp^2^ carbon becomes more dominant. This is concomitant with the significant reduction of heteroatom content (i.e., reduction of C–N, C–O, and C–O–C peaks), especially with decreased relasing speed *v*_2_. Taken together, the results from Raman spectroscopy, XRD, and XPS demonstrate clearly that relasing drives more graphitization, defect healing, and heteroatom reduction, leading to the growth of crystalline graphene domains with less stacking disorder.

To get more insight into the molecular structure of LINC, TEM imaging is conducted to confirm the effect of relasing on the size and quality of the graphitic domains, as shown in Figure 5a–d. TEM images in Figure 5a,b show that LINC lased at *v*_1_ = 105 mm/s shows small graphitic domains around *L*_c_ = 3 nm that are highly disordered with many defects. Figure 5c,d demonstrates the effect of relasing with larger and more ordered graphitic domains having larger *L*_c_ up to 9.3 nm in size and better-stacked graphene layers. These results support the observations from Raman spectroscopy, XRD, and XPS. Overall, the structural and chemical characterization shows that relasing produces larger, more ordered, and less heteroatom-rich graphitic domains, which is consistent with the measured reductions in electrical resistivity and the electrochemical impedance (shown below).

**Figure 5:**
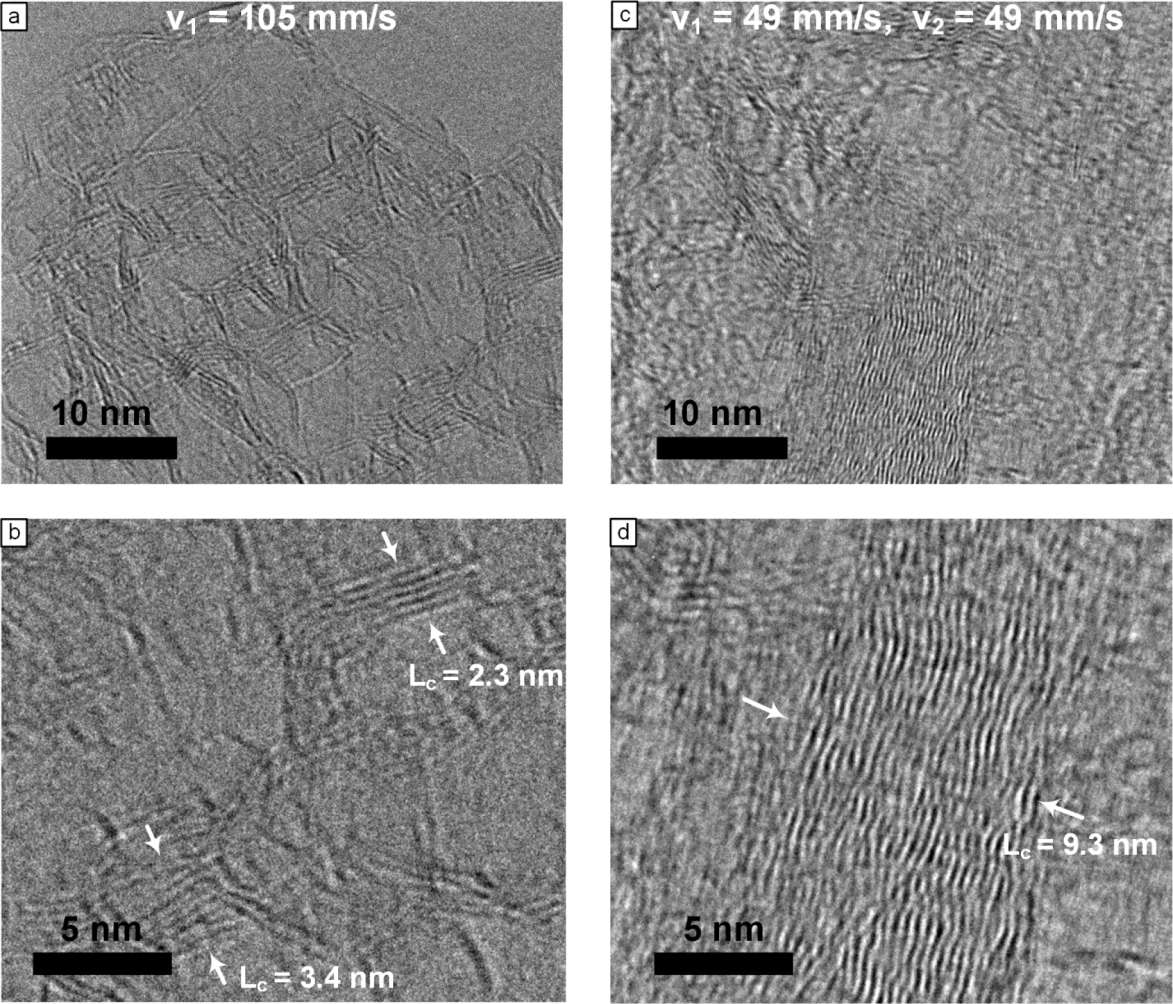
(a) TEM image of LINC at *P* = 12.5 W, *z* = 9 mm, and *v*_1_ = 105 mm/s lasing conditions. (b) HRTEM image showing different *L*_c_ sizes at *v*_1_ = 105 mm/s condition. (c) TEM image of LINC at *P* = 12.5 W, *z* = 9 mm, and *v*_1_ = 105 mm/s and *v*_2_ = 49 mm/s lasing conditions. (d) HRTEM image showing *L*_c_ size at *v*_1_ = 49 mm/s and *v*_2_ = 49 mm/s condition.

### Laser Heating Simulations Confirming Effect of Second Lasing Pass

To further establish the thermal origin of the structural and electrical improvements induced by relasing, we performed finite-element (FE) laser-heating simulations to compare the transient thermal histories of the first- and second-lase steps. Figure 6a shows the three-dimensional temperature field predicted immediately after the second lasing pass, in which the Gaussian moving heat source is applied not to bare polyimide but to a preformed LINC layer with higher absorptivity, higher thermal conductivity, and lower heat capacity relative to PI.^5,52^ The mesh density used in the simulations (inset of Figure 6a) was refined in the region directly beneath the beam path to capture the steep through-thickness and lateral temperature gradients associated with rapid LINC heating. More details about the FE model are included in SI Figures S12 and S13.

**Figure 6:**
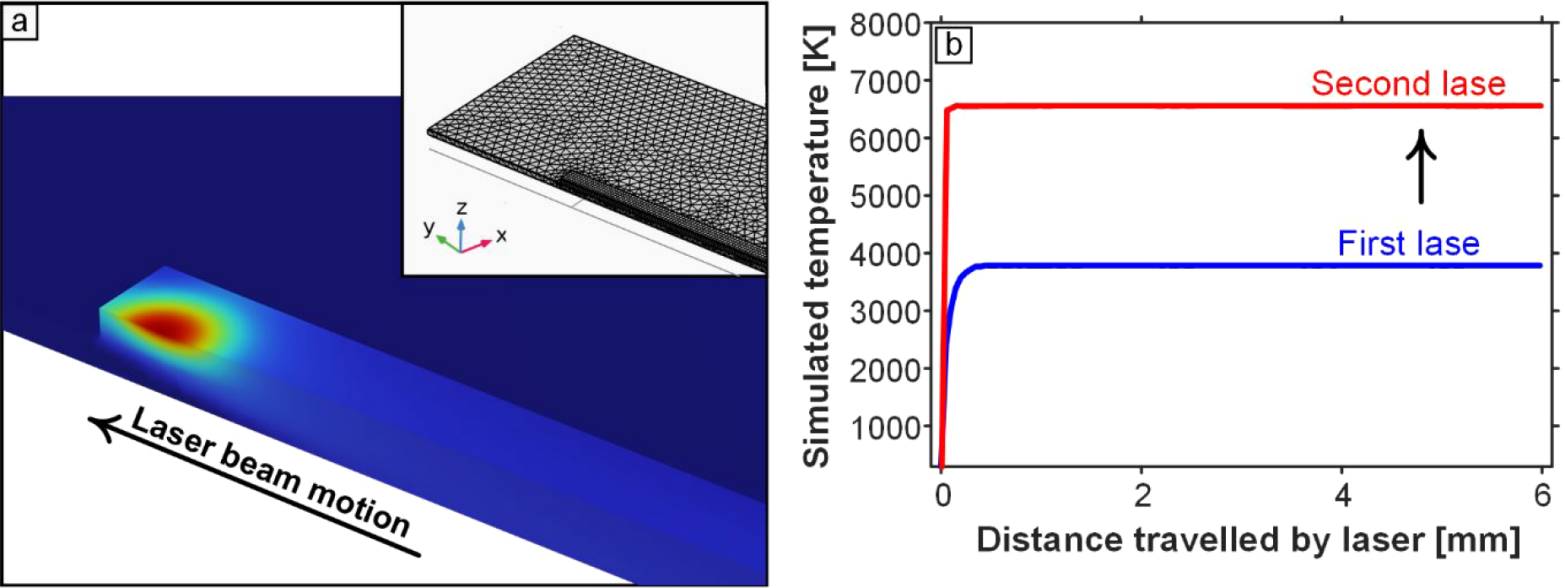
Results of laser heating FEM simulations. (a) Three-dimensional contour plot of temperature distribution after the second lase, considering the material properties of LINC resulting from the first lase. (Inset) Mesh of the PI film in these FEM simulations, showing the finer mesh near and at the heated strip. (b) Simulated temperatures for both the first lase and second lase steps, showing the significant rise in temperature in the second lase (energetically confirming the thermal origin of the increased graphitization and observed ablation).

As shown in Figure 6b, the simulated peak temperature during the second lasing pass is substantially higher than in the first pass, even though the laser power (*P* = 12.5 W), defocus (*z* = 9 mm), and scan speed (49 mm s^–1^) are identical. This amplified heating arises directly from the fact that the incident beam encounters a carbonized LINC surface whose absorption coefficient is significantly larger than that of virgin polyimide at a 10.6 μm wavelength, leading to a higher fraction of the laser energy being converted to heat. Moreover, the LINC layer exhibits a lower thermal diffusivity than PI, which limits heat dissipation into the substrate and results in a more spatially confined high-temperature region. The combination of enhanced absorption and reduced heat spreading produces both a higher maximum temperature during the second lase and a broader region sustaining temperatures above the carbonization–graphitization threshold and even exceeding the ablation threshold.^52^ These features are evident in the contour temperature maps, which show a deeper and wider thermal profile during the second pass.

Importantly, the magnitude of the predicted temperature rise during relaxation is fully consistent with the experimentally observed structural transitions and enhanced conductivity shown in Figures 1–5. Specifically, the higher peak temperatures provide an energetically grounded mechanistic explanation for the following: (1) increased graphitization, reflected in the reduced Raman *I*(D)/*I*(G) ratio, sharper 2D peaks, larger *L*_c_ values from XRD, reduced *d*_002_ spacing, and more ordered multilayer domains observed in TEM; (2) enhanced ablation and crater formation, as captured in the SEM cross sections, which show that the second lasing step removes the outermost layer and exposes a high-surface-area cellular network underneath; and (3) reduced effective resistivity, which stems from the thermally driven improvements in crystallinity and reductions in structural defects. In order to complement these findings, we extend the property characterization to include electrochemical impedance, which is also reduced, owing to combined increases in crystallinity and accessible surface area.

### Electrochemical Impedance Spectroscopy of LINC

To evaluate the potential of the relased LINC electrodes for electrochemical biosensors, we investigated the influence of the relasing process on their electrochemical impedance and DA sensitivity. We fabricate a set of electrodes under three distinct laser conditions using a continuous-wave CO_2_ laser (λ = 10.6 μm) on polyimide sheets (Dupont KHN, polyimide thickness: 127 μm). The laser parameters were set to *P* = 12.5 W and *z* = 9 mm. The lasing speeds for the three conditions were as follows: (a) single lase at *v*_1_ = 105 mm/s, (b) single laser at *v*_1_ = 49 mm/s, and (c) relased at *v*_1_ = 49 mm/s, *v*_2_ = 49 mm/s. Three replicates were created for each condition. The electrodes were then insulated to expose only 1 mm of the electrode. The insulation process is described in the methods section and is illustrated in Figure 7a–e. After the electrodes were insulated to control the working electrode area, SEM imaging was used to analyze the electrode surface area. SEM imaging of the electrode areas is presented in SI Figure S14, revealing a range of surface morphologies across the different conditions.

**Figure 7:**
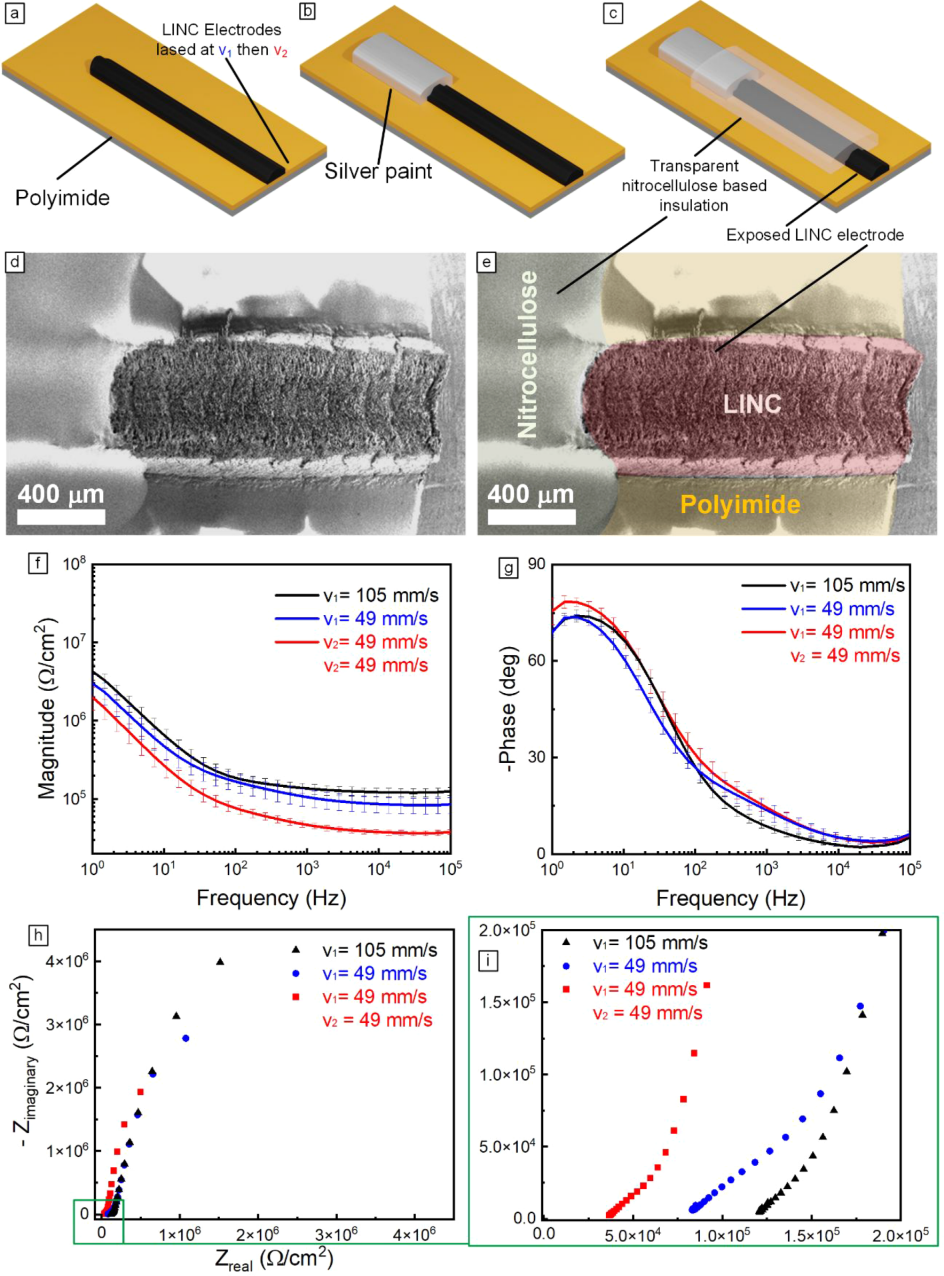
(a,b,c) Schematics illustrating the steps for fabrication and packaging of LINC electrodes for neural sensing applications. (d,e) SEM imaging showing the device packaging with false coloring in (e) to clarify the makeup of the electrode. (f) Plot representing area-normalized averaged EIS results for electrodes created using the different laser conditions. (g) Plot representing area-normalized averaged EIS results for electrodes created using different laser conditions. (h,i) Corresponding Nyquist plots for electrodes fabricated using the different laser conditions, with a high-frequency zoomed-in plot in (i).

The first lasing condition (*v*_1_ = 105 mm/s) produced electrodes with a dome-shaped surface and minimal ablation on the top SI Figure S14a. The second lasing condition (*v*_1_ = 49 mm/s) resulted in electrodes with more pronounced ablation, as evidenced by the presence of a small crater on the top surface (SI Figure S14b). Electrodes fabricated using the third lasing condition (*v*_1_ = 49 mm/s, *v*_2_ = 49 mm/s) exhibited significant ablation, characterized by a large crater at the top of the electrode (SI Figure S14c).

Electrochemical impedance spectra (EIS), presented in Figure 7f,g and Figures S15 and S16, demonstrate that the relasing process notably reduces the electrochemical impedance of LINC electrodes. Overall, the relased LINC electrodes exhibited a lower impedance across the entire 1–10^5^ Hz frequency range. At high frequencies, where capacitive contributions at the electrode–electrolyte interface become negligible, the resistive component of the electrode/solution system can be directly estimated. At 100 kHz, the normalized impedance of the relased electrodes decreases to 37.88 ± 2.91 kΩ/cm^2^ (at *v*_1_ = 49 mm/s, *v*_2_ = 49 mm/s), substantially lower than that of the two single-lase conditions at different speeds (85.57 ± 19.41 kΩ/cm^2^, at *v*_1_ = 49 mm/s and 125.88 ± 14.44 kΩ/cm^2^, at *v*_1_ = 105 mm/s). Consistent with the magnitude results, the Nyquist plots (Figure 7h,i) show a systematic decrease in the high-frequency real-axis intercepts from the higher-speed single-lase condition (*v*_1_ = 105 mm/s) to the lower-speed single-lase condition (*v*_1_ = 49 mm/s), and finally to the double-lase condition (*v*_1_ = 49 mm/s, *v*_2_ = 49 mm/s), indicating a progressive reduction in the uncompensated/ohmic resistance. This trend reflects a progressive reduction in the apparent series (ohmic) resistance. Because the electrolyte conductivity remains constant across measurements and the impedance values are normalized by area, the decrease in real impedance is best attributed to an increase in the intrinsic conductivity of the relased LINC electrode. This enhancement is consistent with ablation-induced increases in surface area, improved graphitization, and modifications in surface chemistry, as described earlier.

In sum, the normalized electrochemical impedance, in the 10^3^–10^5^ Hz range, of the relased electrodes is 2–4 times lower than that of the single-lase electrodes (SI Table S3). Within this frequency range, LINC electrodes exhibit a nearly resistive phase (tending toward 0°), and the impedance modulus is dominated by the combined resistance of the carbon line and the electrolyte.

As further corroborated by the Nyquist plots (Figure 7h,i), the electrode produced under relasing condition (*v*_1_ = 49 mm/s, *v*_2_ = 49 mm/s; red) shows the smallest semicircular arc and the lowest *Z*_real_ values in both the full (Figure 7h) and the high-frequency zoomed-in range (Figure 7i), indicating the lowest charge-transfer resistance (*R*_ct_) and the most efficient electron-transfer kinetics. These results support that the relasing condition yields a more electrochemically active and conductive surface, consistent with improved graphitization or increased effective surface area.

The normalized impedance magnitudes at 1 kHz, physiologically relevant for neural spike recording, are also reported in SI Table S3, showing that the relased electrodes have 2–3 times lower impedance than the single-lase electrodes. We note that the “normalized impedance at 1 kHz” used here is a single-frequency, lumped-cell impedance that includes both solution and electrode contributions and is normalized by the geometric electrode area. Therefore, we complement this metric with two-point and four-point probe measurements in air (Figures 2 and S5) to clearly isolate the electrode-intrinsic conductivity of single-lased and relased LINC electrodes, further demonstrating that the conductivity improvement is intrinsic to the LINC material and is consistently induced by the second lase (“relasing”) step for both line and area LINC electrodes.

Together, these findings demonstrate that the relasing process substantially reduces impedance and enhances the conductivity of LINC electrodes, thereby improving their suitability for electrochemical sensing and neural interfacing applications.

CV conducted in 1x PBS presents an approximately rectangular current response in the −1/1.2 V vs Ag/AgCl potential window (SI Figure S16a), suggesting a predominantly double-layer capacitance-governed response during the charging and discharging process.^48,54^ Only for the relased microelectrodes is it possible to observe a small faradaic oxidation peak at ca. 0.3 V (SI Figure S16 b), similarly to what is observed in highly graphitized graphene electrodes. It is worth noting that LINC microelectrodes show a wide water window, with no hydrolysis reactions occurring between −1 and 1.2 V, making them optimal candidates for electrical microstimulation, where the electrodes are required to inject relatively large currents while minimizing electrode degradation due to faradaic effects. The total charge storage capacity (CSC) values for the LINC obtained with the different laser conditions are reported in SI Table S3. The CSC was calculated as the time integral of an entire CV cycle between −1 and 1.2 V.

### LINC Sensing Performance for the Electrochemical Detection of Tonic Dopamine Levels

Carbon is considered the ideal material for electrochemical detection^35,55–57^ due to its biocompatibility, capacitive electrochemical behavior, and fast electron transfer kinetics. For the last 3 decades, carbon fiber microelectrodes (CFEs) have been used in combination with fast-scan cyclic voltammetry (FSCV)^35^ and are considered the gold standard for the detection of rapid changes in dopamine (DA) concentrations.^35,55^ However, bare CFEs, with low electrochemical surface area, have shown poor sensitivity for resting DA via SWV detection, and a poly­(3,4-ethylenedioxythiophene)/carbon nanotube (PEDOT/CNT) coating was required to increase their sensitivity.^38^ High surface area nanocarbon materials have previously shown significantly higher DA sensitivity than carbon fiber microelectrodes (CFEs) using FSCV^58–61^ but have not been previously used to measure DA resting state.

The sensing performance of LINC for the electrochemical detection of tonic levels of DA was evaluated *in vitro* using a previously optimized SWV,^38^ a pulse voltammetry technique that allows for the isolation of faradic current with an increased peak current amplitude as compared to differential pulse voltammetry.^62,63^

The sensing capability of LINC for DA detection was examined by performing *in vitro* calibration protocols, varying the DA concentrations from 25 nM to 1 μM. LINC electrodes were fabricated at *P* = 12.5 W, *z* = 9 mm, and *v*_1_ = 49 mm/s. These single-lase conditions did not exhibit a linear response to the injection of different DA concentrations and presented high sample variability, as observed by the high standard deviation shown in the error bar in SI Figure S17. Electrodes fabricated at this single-lase condition presented a modest graphitization and surface area, resulting in their capability of DA detection using SWV. The observed variability is attributed to the lack of ablation, which resulted in a nonuniform surface structure with loose nanocarbon flakes on the surface that can be easily removed. In contrast, high levels of ablation for the relased samples resulted in more robust morphology with less sample-to-sample variation, owing to the repeatability and stability of the porous morphology.

On the other hand, LINC electrodes fabricated at *P* = 12.5 W, *z* = 9 mm, and single-lase at *v*_1_ = 105 mm/s lasing conditions, as well as the LINC fabricated at double-lase conditions of *v*_1_ = 49 mm/s, *v*_2_ = 49 mm/s, showed linear responses to DA, as observed from their calibration plots, relating the background-subtracted SWV peak current to DA standard concentration (Figure 8a). They exhibit clear detection of DA at each concentration, with the average SWV traces revealing a single concentration-dependent peak located around 0.12 V (Figure 8b–d). The reduced variability for both the high-speed single-lase and low-speed double-lase conditions highlights the importance of LINC morphology. Contrary to the sample lased at 49 mm/s with loosely held carbon on the surface due to incomplete ablation (Figure 2g), almost no ablation is observed in the case of high-speed single lase (at 105 mm/s), which results in a stable porous morphology and a uniform domed cross-section (Figure 2f). For the double-lased sample (with *v*_1_ = 49 mm/s and *v*_2_ = 49 mm/s), the second lasing step clears the surface of any debris, also resulting in a more stable cratered morphology (Figure 2h).

**Figure 8:**
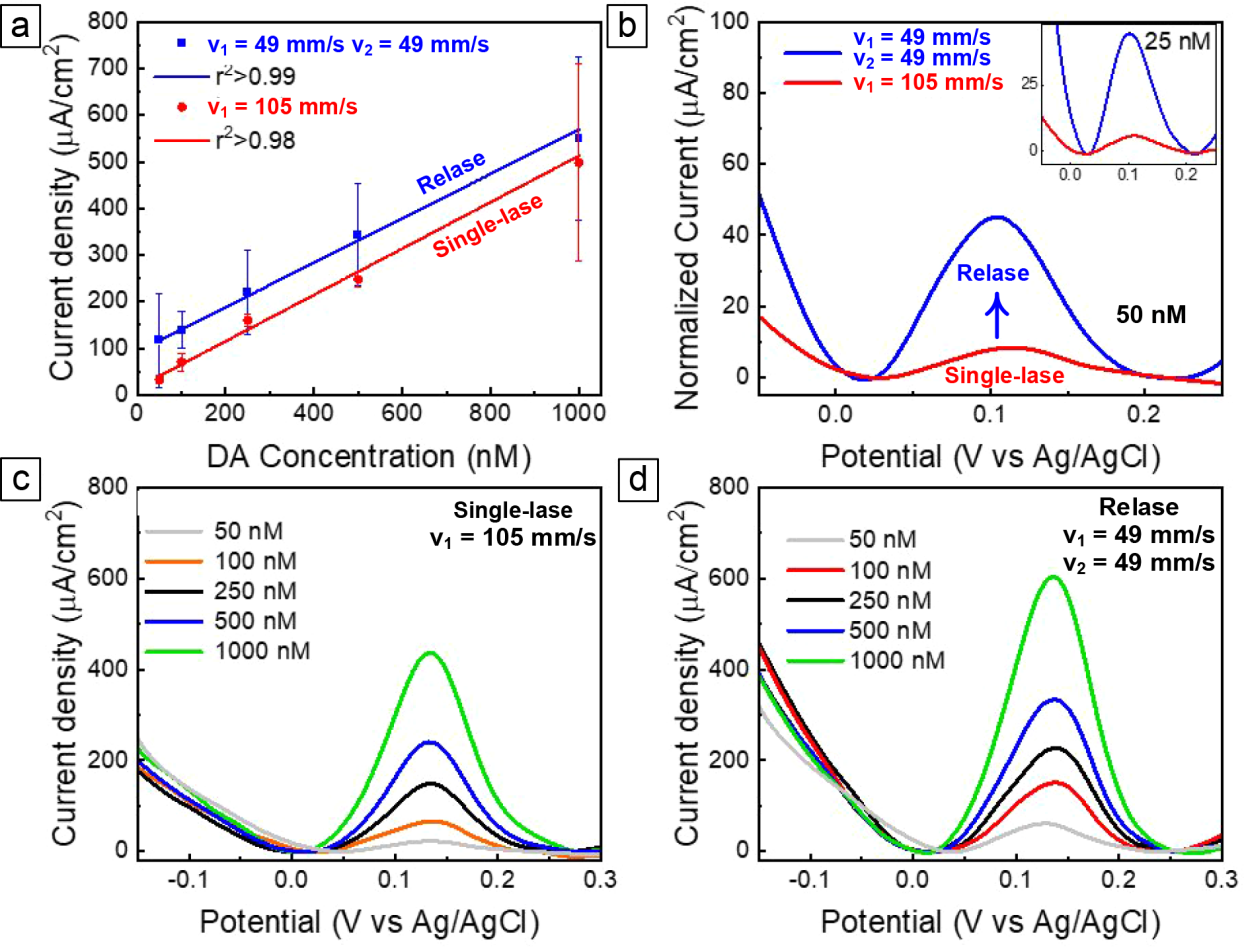
Dopamine sensitivity for LINC electrodes fabricated at *P* = 12.5 W, *z* = 9 mm, and *v*_1_ = 105 mm/s lasing conditions, and *P* = 12.5 W, *z* = 9 mm and *v*_1_ = 49 mm/s, *v*_2_ = 49 mm/s lasing conditions. (a) Calibration curves (10 nM–1.0 μM concentration range) of DA in PBS. (b) Corresponding background-subtracted CVs, highlight the dopamine oxidation peaks at 25 nM and 50 nM DA injection. (c,d) Representative background-subtracted CVs for 50, 100, 250, 500 nM, and 1 μM bolus of DA injection collected.

The electrode sensitivity toward DA detection, defined as the linear slope of the calibration plots (Figure 8a), is 84 ± 8 nA/μM for LINC electrodes fabricated under the single-lase condition (*v*_1_ = 105 mm/s) and 130 ± 5 nA/μM for electrodes fabricated using the double-lase condition (*v*_1_ = 49 mm/s, *v*_2_ = 49 mm/s). After electroactive-area normalization, the sensitivities are 497 ± 21 (μA/cm^2^)/nM and 476 ± 18 (μA/cm^2^)/nM, respectively, which are comparable to those reported for PEDOT/CNT-coated CFEs^38^ and PEDOT/CNT-coated GC-MEAs.^44^ Notably, even when normalized, and even though the slopes of the calibration curves are similar, the LINC electrodes fabricated by a double-lasing step at *v*_1_ = 49 mm/s and *v*_2_ = 49 mm/s can detect higher current peak amplitudes at all the concentrations, enabling a more reliable detection of concentrations as low as 25 and 50 nM (Figure 8b), within the range of the physiological concentrations of resting DA in the brain.^38,64–67^ In agreement with this result, the limit of detection (LOD), determined as three times the standard deviation of the baseline noise divided by the slope of the calibration curve obtained from the linear regression of current versus DA concentration, is 4 nM for relased electrodes and 19 nM for single-lased electrodes.

The improved sensitivity of the relased LINC is driven by the higher surface area morphology of the nanocarbon, compared to the LINC lased at *v*_1_ = 105 mm/s, in combination with a superior graphitization. Since electrochemistry is a surface phenomenon, the high surface area of the nanocarbon materials facilitates DA adsorption and increases sensitivity by concentrating DA onto the electrode surface. Thus, LINC electrodes fabricated by double-lasing at low speeds (*v*_1_ = *v*_2_ = 49 mm/s) provide the increase in sensitivity required to measure DA resting state using SWV. Additionally, uniform graphitization of carbon materials has been associated with notably higher conductivity^68^ and enhanced sensitivity toward DA and other surface-sensitive species, due to the numerous graphitic edges present in the carbon lattice.^69^ The presence of these defects is associated with the quantity of the electroactive sites on the carbon electrodes,^69^ and edge-plane-rich, sp^2^-hybridized carbon nanomaterials have been shown to increase DA detection sensitivity, selectivity, and fouling resistance.^35,58,60^

To our knowledge, although laser-induced graphene (LIG) electrodes have been used previously for fast-scan cyclic voltammetry (FSCV) by our group^25^ and others,^50^ they have not been evaluated using SWV. Thus, this work represents the first demonstration of SWV-based DA detection using LINC electrodes, enabling direct comparison to recent studies employing the same SWV waveform for tonic DA detection (Table 1). The SWV waveform used in this study was specifically optimized for selective DA detection,^38^ while a separate SWV waveform was optimized for serotonin (5-HT) detection.^43^ 5-HT is a cationic indolamine neurotransmitter that, like DA, can be electrochemically oxidized within the physiological pH solvent window. There is significant interest in understanding the interplay between DA and 5-HT release in reward processing and learning, as well as in the progression of neurological diseases.^70^ As a proof of concept demonstrating the ability of LINC to detect both tonic DA and 5-HT simultaneously, we extended the DA waveform and observed distinct DA and 5-HT peaks at 0.15 and 0.30 V vs Ag/AgCl, respectively (Figure S18). Additionally, compared with CFEs, LINC exhibited markedly higher sensitivity toward DA detection (see the inset in Figure S18).

**Table 1:** DA Sensing (Tonic Level) Using CFE and Carbon-Based Microelectrode Arrays (MEAs) Using SWV^a^

Electrode	DA SWV sensitivity	Electrode size	DA range tested	From
Full glassy carbon fibers (fGCFs)	0.038 ± 0.001 μA/nM μm^2^	10 μm wide,10 μm thick 100 μm long	50 nM–1 μM	Siwakoti et al.^71^
Glassy carbon fiber-like (GCF)-MEA	0.45 μA cm^–2^ nM^–1^	10 μm wide, 200 μm long Double sided	25 nM–200 nM	Castagnola et al.^46^
CFEs	0.33 μA cm^–2^ nM^–1^	7 μm diameter 400 um length	25 nM–200 nM	Castagnola et al.^46^
PEDOT/CNT-coated silicon MEA	0.0147 ± 0.0005 nA/μM	1200 μm^2^ gold electrode sites	100 nM–1 μM	Taylor et al.^38^
PEDOT/CNT-coated CFEs	90 ± 7 nA/μM	7 μm diameter 400 um length	100 nM–1 μM	Taylor et al.^38^
PEDOT/CNT-coated flexible MEA	0.15 nA/nM	35 μm diameter PEDOT/CNT-coated Pt- microelectrodes	50 nM–500 nM	Wu et al.^72^
PEDOT/CNT-coated flexible GC-MEA	55.634 ± 0.001 nA/μM	40 μm diameter PEDOT/CNT-coated GC microelectrodes	10 nM–1 μM	Castagnola et al.^44^
Flexible GC-MEA	10.22 ± 0.33 nA/μM	40 μm diameter bare GC microelectrodes	10 nM–1 μM	Castagnola et al.^44^
LINC single-lase at *v*_1_ = 105 mm/s	84 ± 8 nA/μM	500 μm wide, 1.5 mm long	10 nM–1.0 μM	*This work
LINC double-lase at *v*_1_ = 49 mm/s, *v*_2_ = 49 mm/s	130 ± 5 nA/μM	600 μm wide, 1.5 mm long	10 nM–1.0 μM	*This work

a Asterisk denotes current study. Acronyms used: GC—glassy carbon, CFEs—carbon fiber microelectrodes, MEAs—microelectrode arrays, CNT—carbon nanotubes, PEDOT/CNT—poly­(3,4-ethylenedioxythiophene)/CNT, DA—dopamine, SWV—square wave voltammetry, fGCfs—full glassy carbon fibers, GCF-MEA—glassy carbon fiber-like (GCF)-MEA.

In this work, all DA sensing measurements should be interpreted as a benchtop demonstration of a LINC proof-of-concept for the DA sensing performance. These measurements will require further evaluation of stability, selectivity, and biofouling, both *in vitro* and *in vivo*, in future studies. Although additional miniaturization will be necessary before implantation and *in vivo* neurochemical measurements, these findings clearly show that SWV measurements using LINC electrodes enable strong peak responses and highly sensitive detection of low DA concentrations.

### Electrochemical Stability, Mechanical Robustness, and Manufacturing Scalability

We also evaluate the effect of accelerated aging on relased LINC electrodes through prolonged biphasic pulsing in phosphate-buffered saline (PBS). Figure 9 shows the impedance spectra before and after 120 million, 680 million, and 1 billion cycles of stimulation pulses in PBS (corresponding to 360 h of stimulation). Importantly, we show that these LINC electrodes exhibit exceptional electrochemical stability under aggressive electrical stimulation. In these tests, we applied continuous cathodic-first charge-balanced 500 μs biphasic current pulses with 6.5 mA of amplitude, with a charge density corresponding to 1/2 the charge injection limit of 2.8 mC/cm^2^. At regular intervals, impedance spectra were collected, and the integrity of the electrodes was checked. Even after 1 billion cycles, we do not see significant changes in impedance to indicate any failure in the LINC electrodes. For example, we do not see any abrupt increase in impedance that would be indicative of cracking, delamination, or flaking of the LINC electrodes. These findings underscore the unique mechanical robustness and strong substrate adhesion of our porous LINC on the polyimide substrate.

**Figure 9:**
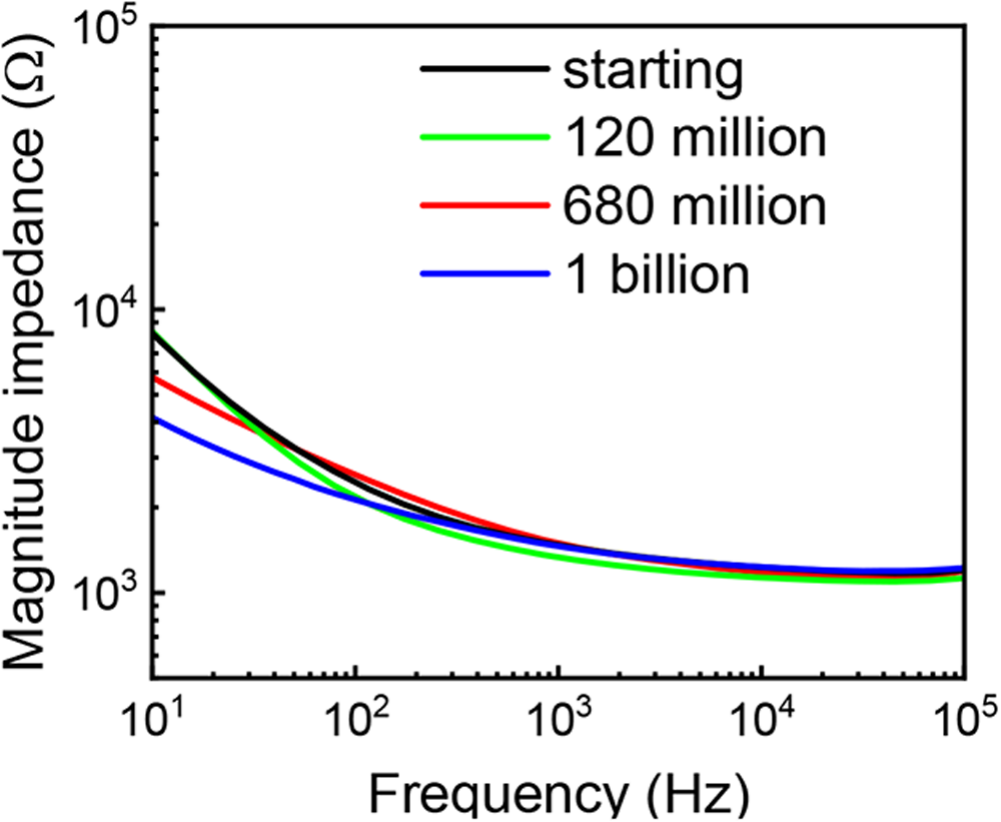
Results for aggressive electrochemical stability evaluation showing the electrochemical impedance spectra (EIS) of the relased LINC electrode up to 1 billion cycles of biphasic pulse stimulation.

To further demonstrate the long-term performance and mechanical robustness of LINC area electrodes on flexible devices, we carried out cyclic bending tests (Figure S19), which show that the normalized sheet resistance (*R*/*R*_0_) changes by less than ∼11% after 2000 bending cycles to a bend radius of 4 mm. These results highlight the stable electrical performance under repeated mechanical deformation on the flexible substrate, which emphasizes the potential of our approach for implantable and wearable sensors. This consistency in electrical resistance and absence of visible cracking or peeling depicted in microscopy images before and after 2000 bending cycles suggest good adhesion of LINC to the substrate. Overall, our results indicate that relased LINC electrodes are promising for future implant studies and for potential miniaturization into neural microelectrodes for applications such as neurochemical sensing, electrical microstimulation, and electrophysiological recording, although comprehensive long-term stability, biocompatibility, and encapsulation strategies remain to be established.

An important advantage of the sequential irradiation strategy demonstrated in this work is that it is inherently compatible with scalable and cost-effective manufacturing. Because the entire process relies on direct laser writing of commercial polyimide films using a single CO_2_ laser head in air, electrode geometries are defined purely by programmed scan paths without the need for cleanroom lithography, masks, vacuum processing, wet chemistry, or postprocessing additives. This mask-free approach allows both high-resolution single-line electrodes and large-area arrays to be produced by using the same tool by switching between vector and raster writing modes, offering straightforward scalability to roll-to-roll or gantry-based manufacturing platforms. The sequential lasing (“relasing”) step also requires no additional hardware or materials, as it simply reuses the same optical path with an adjusted scan speed. This makes the performance gains demonstrated here achievable without increasing the system complexity or cost. In conjunction with prior studies establishing LIG/LINC as a scalable, industrially relevant process, our results confirm that the enhanced graphitization, reduced resistivity, and improved sensing performance obtained through relasing can be realized in manufacturing environments with minimal overhead, positioning this method as a practical route toward high-volume production of flexible biosensors and neural interfaces.

## Conclusions

Using a sequential irradiation approach, we enhanced the electrical conductivity and reduced the electrochemical impedance of LINC electrodes without integrating additives. The influence of the lasing and relasing speed on the graphitization, crystallinity, and surface morphology/chemistry has been elucidated. We demonstrate that using relasing, LINC’s effective conductivity can be enhanced to 0.08 Ω·cm without excessive electrode damage owing to improved graphitization. Additionally, by leveraging LINC ablation, the surface porosity of LINC can be controlled by relasing. By complementing our experiments with finite element simulations of laser heating, the coupled thermal–structural picture that emerges is that the first lase primarily carbonizes the PI, creating a porous but relatively disordered LINC scaffold with modest thermal absorption. The second lase then interacts with this carbonized layer to reach substantially higher local temperatures, enabling defect healing, sp^2^ domain growth, heteroatom removal, and controlled ablation. These thermally driven changes provide a direct mechanistic explanation for the strong speed dependence, the morphology transitions, and the significant improvement in conductivity and sensing performance documented in this work. Hence, our work sheds light on the fundamentals of LINC formation fabricated using continuous-wave CO_2_ lasers on polyimide films. Importantly, our relased electrodes demonstrate a significantly reduced detection limit of dopamine at very low concentrations below 25 nM, owing to the combination of high surface area and improved conductivity enabled by relasing. Combined with the long-term electrochemical stability, mechanical robustness, and the possibility of heteroatom-doping of LINC, this approach is promising for not only electrochemical sensing but also neural stimulation and recording, which paves the way for advanced neural probes with multifunctionality/multimodality.

## Experimental Section

### LINC Formation on Polyimide

Two types of polyimide tape were used in experiments: TAPECASE 2B (Cat. No. 15C616, polyimide thickness: 50 μm) and Dupont KHN (polyimide thickness: 127 μm) as substrate precursors for LINC formation experiments. To prepare for the experiments, the tape was placed on silicon wafers, rinsed with acetone, and then isopropyl alcohol for sample surface cleaning. Direct laser irradiation on the polyimide sheets was conducted in air using a continuous-wave CO_2_ laser cutter/engraver system (Full Spectrum Laser Pro-Series 20 × 12, 1.5 in. focus lens) with a 10.6 μm wavelength and 45 W power. The system allows tuning of the power by controlling the laser current. We measure the laser power at different currents using a CO_2_ laser power meter (HLP-200, Changchun Laser Optoelectronics Technology Co., Ltd.). The beam radius was measured based on (1/e^2^) of the maximum intensity (w_*y*_, w_*x*_) at different distances (*z*) from the beam waist using the knife-edge method. Using this technique, the beam radius at the beam waist (w_ox_, w_oy_), based on a Gaussian beam assumption, was determined to be 125.8 μm in the *x*-direction and 84 μm in the *y*-direction. The laser objective lens is mounted on a motorized XY stage with a maximum speed of 500 mm/s in the *X*-direction. The beam spot size was controlled by adjusting the vertical distance (*z*) between the sample position and the beam waist; i.e., by moving the sample stage vertically with respect to the objective lens. A defocus level of *z* = 9 mm, corresponding to a spot size of *w* = 500 μm, is used in all experiments. Lasing speed (*v*) was varied from 29 to 250 mm/s, and experiments were performed at a power *P* = 12.5 W and under ambient conditions.

### Characterization of LINC

SEM images of the LINC formations were taken on a Zeiss SIGMA VP field emission scanning electron microscope. The samples were sputter-coated with platinum and then imaged with a beam with an accelerating voltage of 2 kV. An XplorA Raman-AFM/TERS system microscope using 473 nm laser excitation at room temperature with a laser power of 25 mW was employed to obtain Raman spectra. XPS analysis was performed using a Thermo Fisher ESCALAB 250 Xi XPS at a base pressure of 5 × 10^–9^ Torr. All of the survey spectra were recorded in a 1 eV step size. Elemental core spectra were recorded in 0.1 eV step sizes. All of the spectra were corrected using C 1s peaks (284.5 eV) as references. The TEM imaging was performed using a Hitachi H-9500 E-TEM. The LINC electrodes were scraped off and sonicated in ethanol before being drop cast onto a TEM grid (Lacey Carbon, 200 mesh, Ted Pella, Inc., 01881) and then imaged. X-ray diffraction (XRD) was conducted using a Bruker D8 Discover SRD X-ray diffractometer with Cu Kα radiation (λ = 1.54 Å). The LINC electrodes were scraped off and placed on a silicon crystal zero diffraction plate (MTI, ZeroSi24D10C1US). The resistance of the LINC lines was measured using a Keithley two-point probe meter (model: 2100, detection limit: 100 MΩ). LINC lines of lengths of 15 mm are lased in the polyimide at different *z* values. A nickel paste (PELCO Conductive Nickel Paint, Ted Pella, Inc., 16062) was applied to the LINC lines at the measurement spot areas for better contact with the probe terminals. The nickel paint was allowed to dry overnight in air before measurements. The resistivity of LINC was measured and averaged, and the cross-sectional area is measured to estimate effective resistivity. To characterize the resistivity of two-dimensional LINC areas, square LINC pads (12.7 mm × 12.7 mm) are fabricated using *P* = 12.5 W, speed = 111 mm·s^–1^, *z* = 9 mm, and raster gap = 355 μm for both single-lased and relased cases. The sheet resistance of these pads was measured by using a four-point probe via the van der Pauw method.

### Finite Element Simulation of First- and Second-Lase Heating

A transient finite element heat-transfer model was implemented in COMSOL Multiphysics (Heat Transfer in Solids module) to compare the thermal histories during the first- and second-lase steps. In the first-lase case, a moving Gaussian surface heat flux was applied to a PI substrate, whereas in the second-lase case, the same laser parameters (*P* = 12.5 W, speed = 49 mm/s, *z* = 9 mm) were applied while scanning over a preformed LINC electrode on PI. Convection boundary conditions were imposed on the top and bottom surfaces, and the top surface additionally included surface-to-ambient radiation. Temperature-dependent thermal properties were used for PI based on values from literature and our recent work.^5,52^ The model geometry, mesh, boundary conditions, and full implementation details are provided in Figures S12 and S13 and the accompanying text in the Supporting Information.

### Fabrication of LINC-Based Neural Sensors

LINC electrodes are fabricated by lasing single lines at *P* = 12.5 W and *z* = 9 mm with *v*_1_ or *v*_1_ and then *v*_2_. Nitrocellulose-based transparent nail polish (7417045109) is applied as an insulation layer to define the working electrode area, which is controlled to be a specific length according to the testing method. This specific length is achieved by trimming the exposed electrode after applying the insulation layer so that the exposed length is identical for all samples (Figure 7). Because the electrode width is set by a single laser-written line, this ensures that the exposed electrode area is effectively identical across samples within a given set of parameters. The electrodes were externally connected with conductive flat-jaw alligator connections that clamp onto silver paint (PELCO Conductive Silver Paint, Ted Pella, Inc. 16062) applied to the end of the electrode. The silver paste and alligator connection are also insulated with the nitrocellulose-based insulation.

### Neural Probe Testing of LINC Electrodes

Two electrochemical testing methods were used to test the electrodes: Electrochemical Impedance Spectroscopy (EIS), to determine the electrical properties of the system over a large frequency range, and Cyclic Voltammetry (CV), to quantify the electrode capacitive charging. Electrochemical Impedance Spectroscopy (EIS) was performed in 1× phosphate-buffered saline (PBS, composition: 11.9 mM Na_2_HPO_4_ and KH_2_PO_4_, 137 mM NaCl, 2.7 mM KCl, pH 7.4) by applying a sine wave (10 mV RMS amplitude) onto the open circuit potential while varying the frequency from 1 to 10^5^ Hz. EIS was carried out using a potentiostat/galvanostat (Autolab, Metrohm, USA) connected to a three-electrode electrochemical cell with a platinum counter electrode and a Ag/AgCl reference electrode. During the CV tests, the working electrode potential was swept between 1.2 and −1 V (vs Ag/AgCl) with a scan rate of 100 mV/s. The charge storage capacity (CSC, mC/cm^2^) was calculated as CSC = (∫di dt)/(geometric area) in an entire CV cycle.

### Neurotransmitter (Dopamine) Detection

The sensing performance of the LINC for the electrochemical detection of tonic levels of DA was evaluated *in vitro* using a previously optimized SWV,^38^ a pulse voltammetry technique that allows for the isolation of faradic current with an increased peak current amplitude as compared to differential pulse voltammetry.^62,63^ The SWV waveform was repeatedly applied from −0.2 to 0.3 V with a 25 Hz step frequency, a 50 mV pulse amplitude, and a 5 mV step height every 15 s. The potential was held at 0 V between scans. The sensing capability of LINC for DA detection was examined by performing *in vitro* calibration protocols, varying the DA concentrations from 25 nM to 1 μM.

## Supplementary Material



## Notes

Illustration of beam shape; SEM images showing effect of relasing; SEM images showing electrode cross-sections; SEM images illustrating pore thickness; plot of relasing speed vs Raman *I*(2D)/*I*(G) ratio; plots of XRD deconvolution; bar plots illustrating influence of lasing speed and relasing on crystal size, spacing, and number of layers; XPS survey scans; C 1s core scans; SEMs illustrating neural probe packaging; plots of electrochemical impedance spectroscopy; plot representing area-normalized averaged CV results; SWV sensitivity of tonic dopamine (DA) concentrations; table of XRD deconvolution; table of C 1s deconvolution; table of average impedance and CSC values; more experimental measurements of resistivity for relased LINC lines and areas, along with proper statistics; bending test results demonstrating the long-term stability of the resistivity of LINC electrodes; modeling details and results for the finite element simulations of laser heating (PDF)
