# Supplementary material for: Lowering Impedance and Improving Sensitivity in Laser-Induced Graphene Biosensors via Speed-Dependent Sequential Irradiation
Source: ACS Appl Mater Interfaces. 2025 Dec 29;18(1):613–28. doi: 10.1021/acsami.5c20377 (PMC12781063; doi:10.1021/acsami.5c20377)
Supplement: Supplementary file 1 [file am5c20377_si_001.pdf]

Supporting Information

Lowering Impedance and Improving Sensitivity in Laser-Induced Graphene Biosensors via Speed-Dependent Sequential Irradiation

Moataz Abdulhafez,[1,2] Elisa Castagnola,[3,4,5,6] Mirza Sahaluddin,[1] Giulia Baglieri,[3] Golnaz N. Tomaraei,[1,2] Soumalya Ghosh,[1] Xinyan Tracy Cui,[3] and Mostafa Bedewy.[1,2,7,*]

[1] Department of Mechanical Engineering and Materials Science, University of Pittsburgh, 3700 O'Hara Street, Pittsburgh, PA 15261, USA

[2] Department of Industrial Engineering, University of Pittsburgh, 3700 O'Hara Street, Pittsburgh, PA 15261, USA

[3] Department of Bioengineering, University of Pittsburgh, 3700 O'Hara Street, Pittsburgh, PA 15261, USA

[4] Department of Biomedical Engineering, Louisiana Tech University, Ruston LA 71272

[5] Institute for Micromanufacturing, Louisiana Tech University, Ruston LA 71272

[6] Department of Neurosurgery, Louisiana State University Health Sciences, Shreveport LA 71103

[7] Department of Chemical and Petroleum Engineering, University of Pittsburgh, 3700 O'Hara Street, Pittsburgh, PA 15261, USA

*author to whom correspondence should be addressed.

E-mail address: mbedewy@pitt.edu (M. Bedewy)

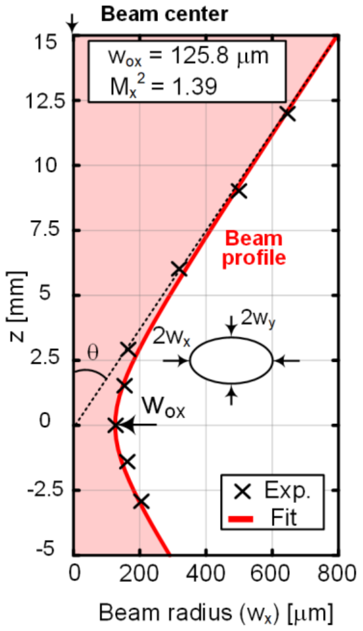

Figure S1. Plot illustrating the experimentally estimated shape of the laser beam as a function of laser beam defocus (z) used in this study.

.

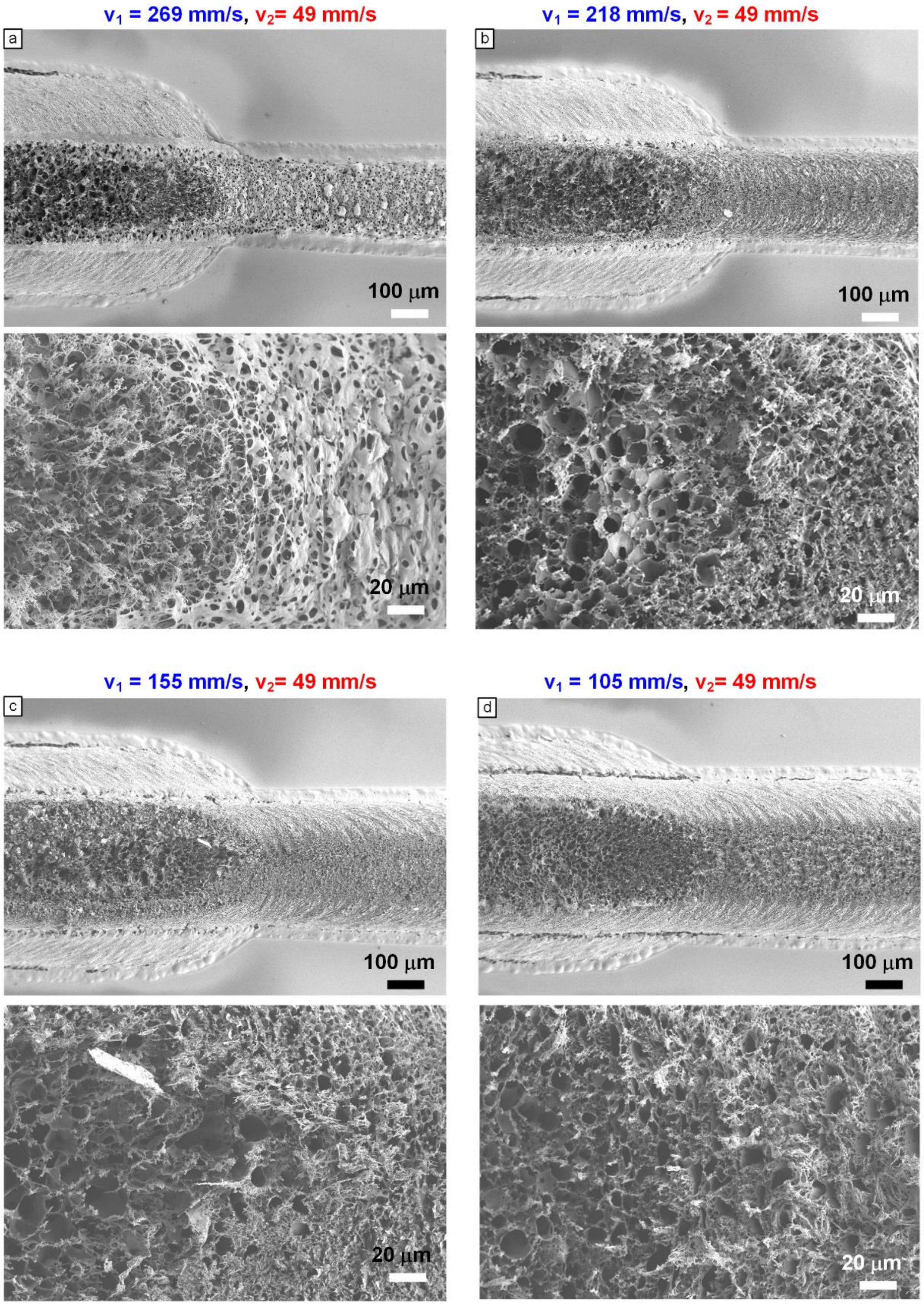

Figure S2. SEM images of LINC lines lased at P = 12.5 W, z = 9 mm (a) v_1 = 269 mm/s, (b) v_1 =218 mm/s, (c) v_1 = 155 mm/s and (d) v_1 = 105 mm/s and then relased at v_2 = 49 mm/s showing the interface between the single-lased segment and relased segment.

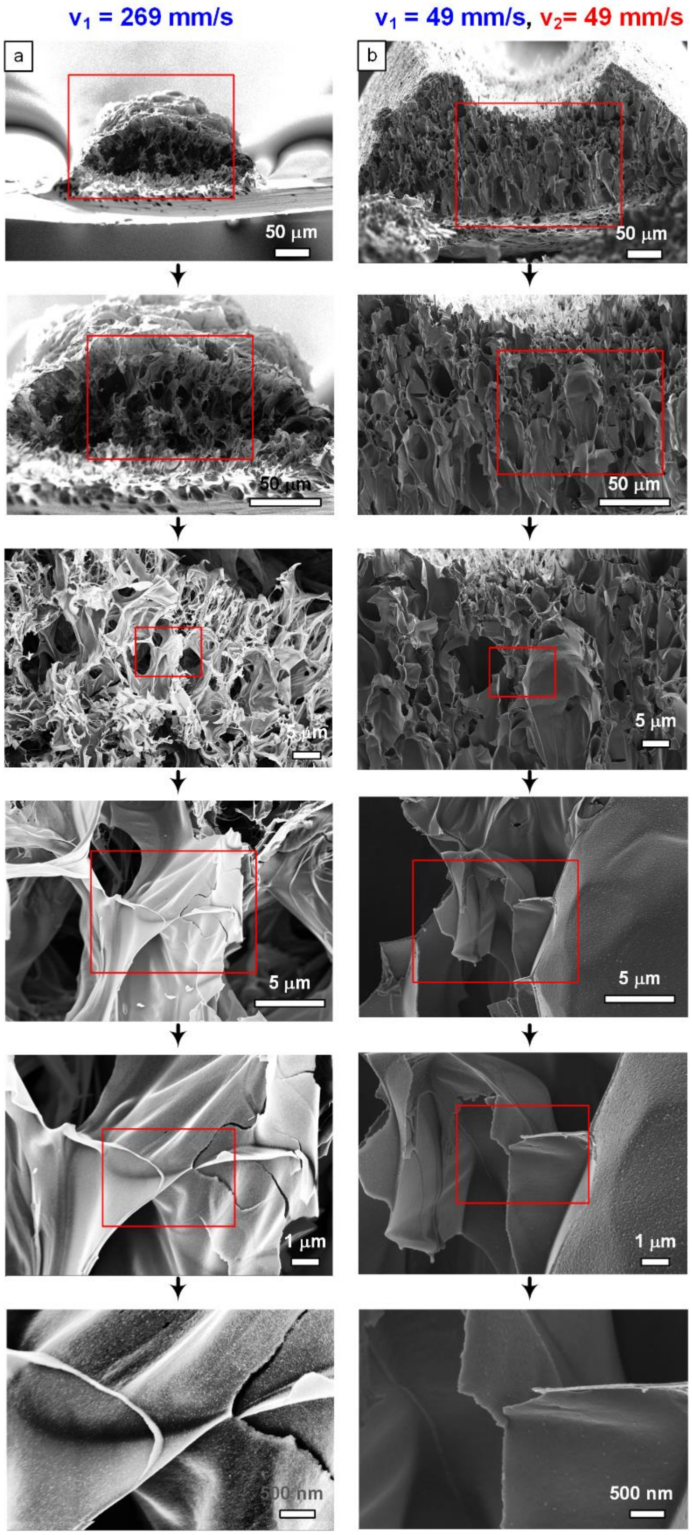

Figure S3. SEM showing mechanically fractured LINC lines lased at conditions (a) v_1 = 269 mm/s and (b) v_1 = 49 mm/s, v_2 = 49 mm/s illustrating the inner structure of LINC lines.

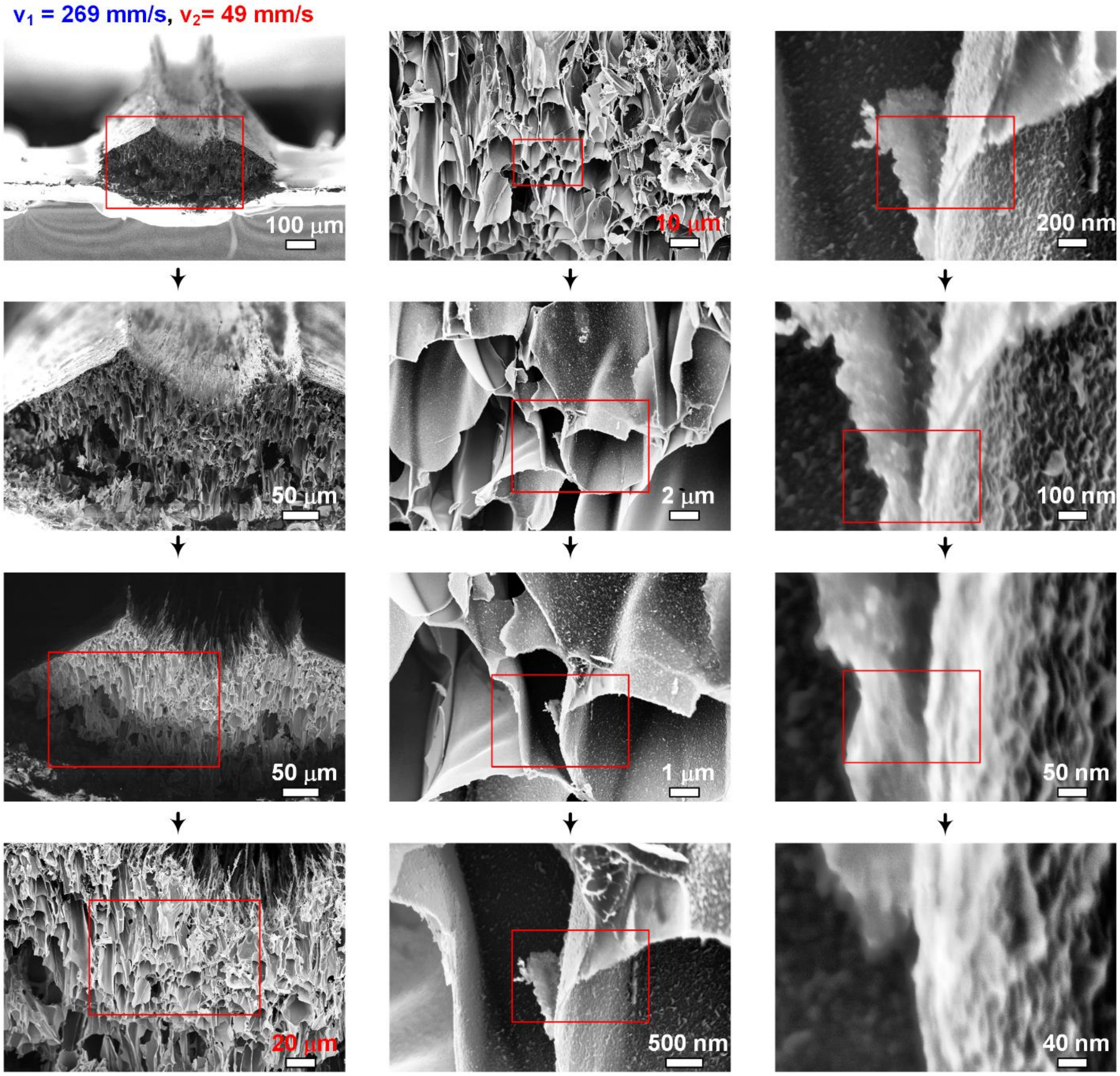

Figure S4. SEM showing mechanically fractured LINC lines lased using conditions P = 12.5 W v_1 = 269 mm/s and v_2 = 49 mm/s illustrating the inner structure of LINC lines and the nanoscale thickness of the inner pore walls.

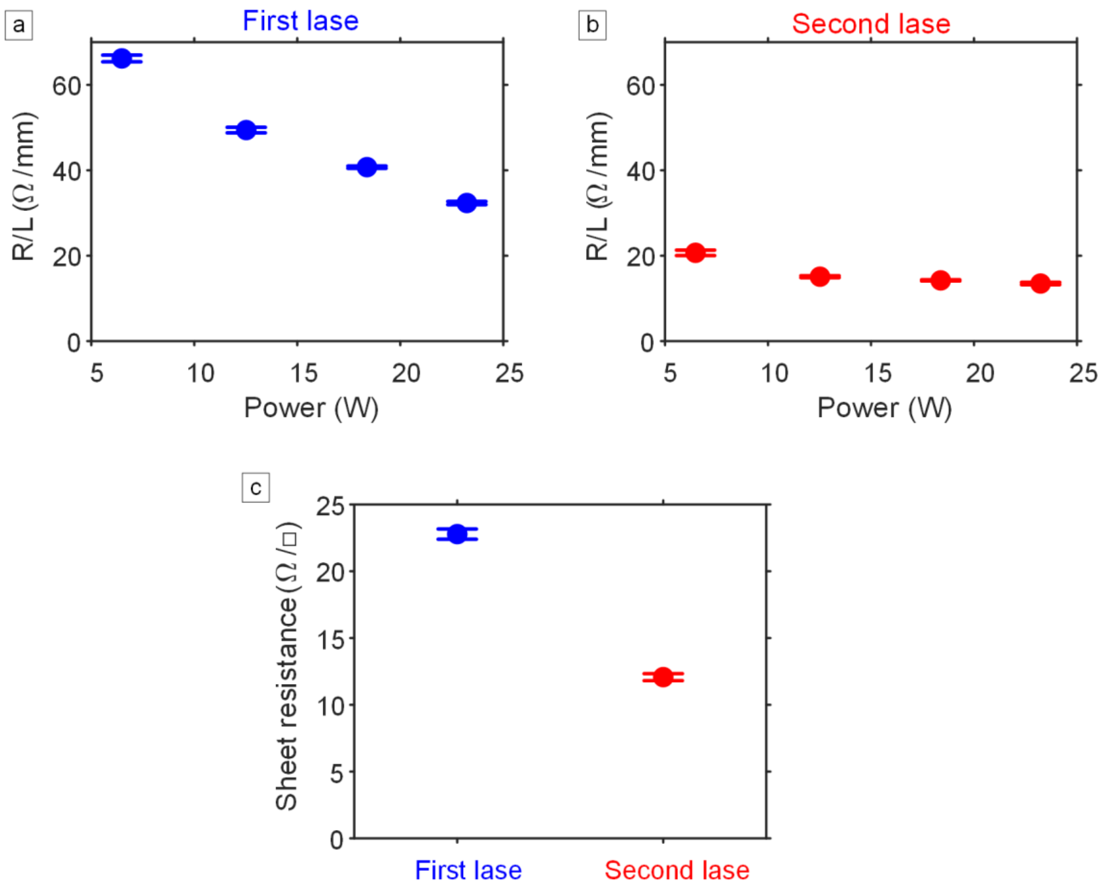

Figure S5. Resistance per unit length measured at different powers for (a) single-lased electrode lines (speed = 111 mm/s and z = 9 mm) and (b) relased electrode lines (speed = 111 mm/s and z = 9 mm) with their corresponding applied powers. (c) Sheet resistance measured using the van der Pauw method for electrode areas fabricated with the first and second lase, showing the reduction in sheet resistance after the second lase (P = 12.5 W, speed = 111 mm/s, z = 6 mm, and raster gap = 355 µm). Each data point represents n = 3 independently fabricated samples, and the error bars indicate the standard error, which is shown to be small, supporting the repeatability and consistency of results.

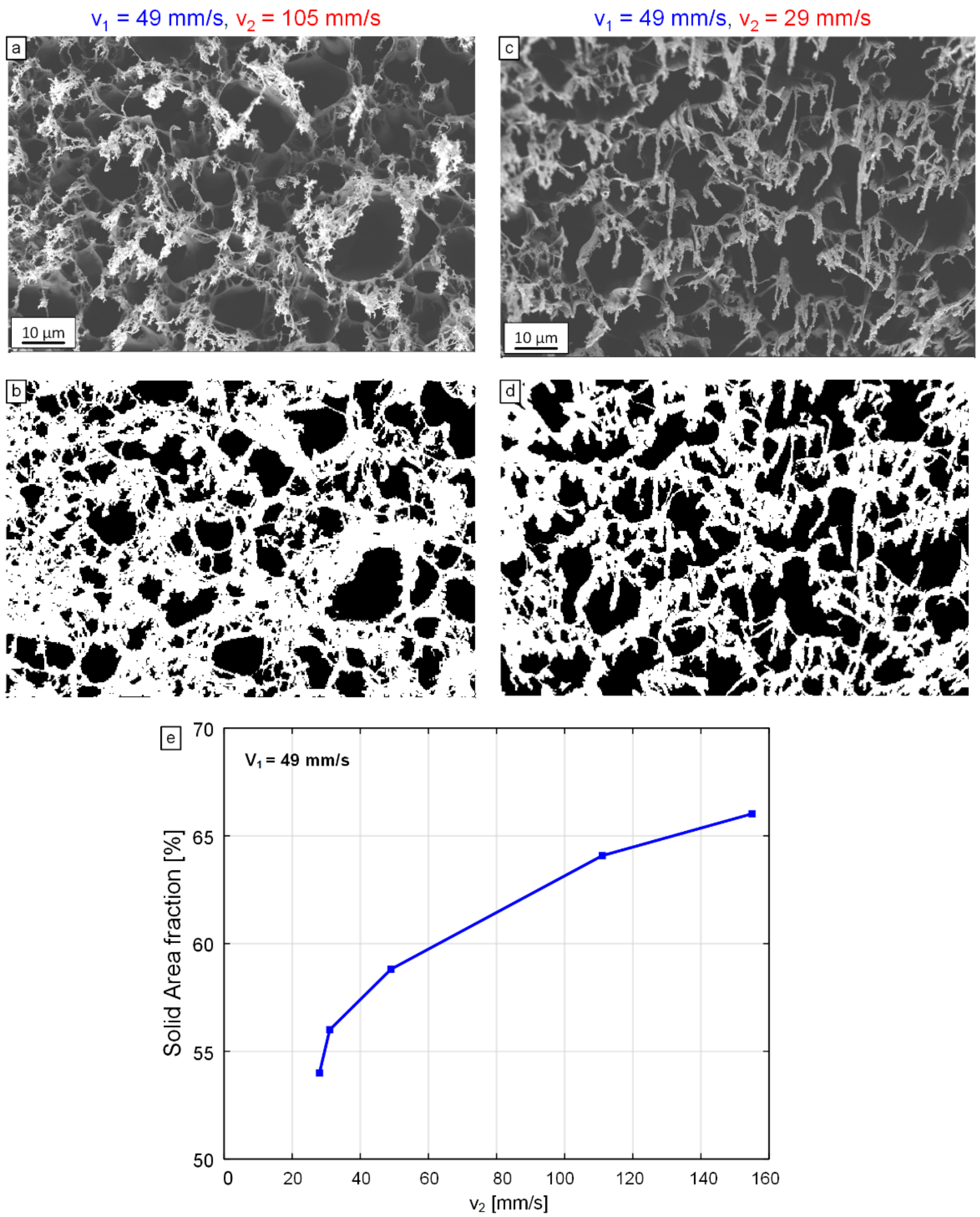

Figure S6. (a,d) SEM images and binary images illustrating the influence of relasing on the surface mesoscale porosity of LINC. (e) Plot generated from binary image processing analysis illustrating the potential of porosity control using relasing.

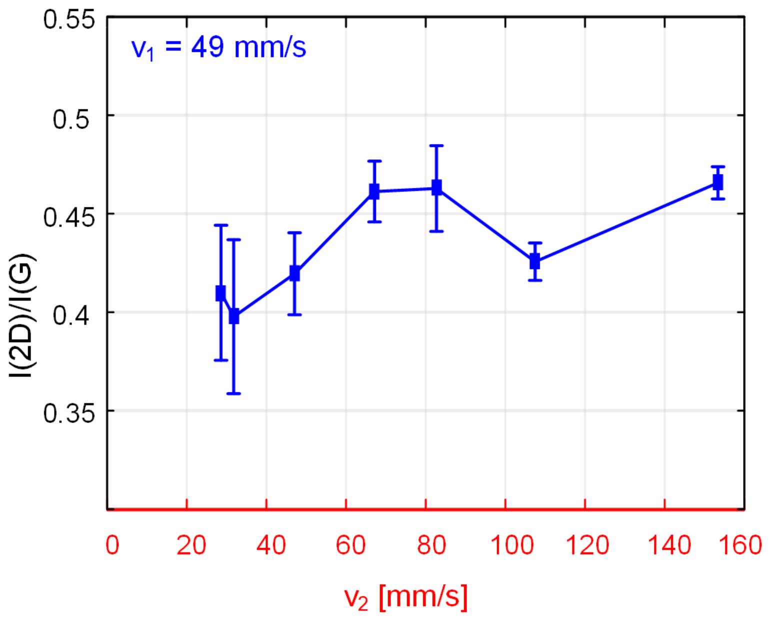

Figure S7. Plot of I(2D)/I(G) of LINC lines lased using conditions P = 12.5W, z = 9 mm, v_1 = 49 mm/s.

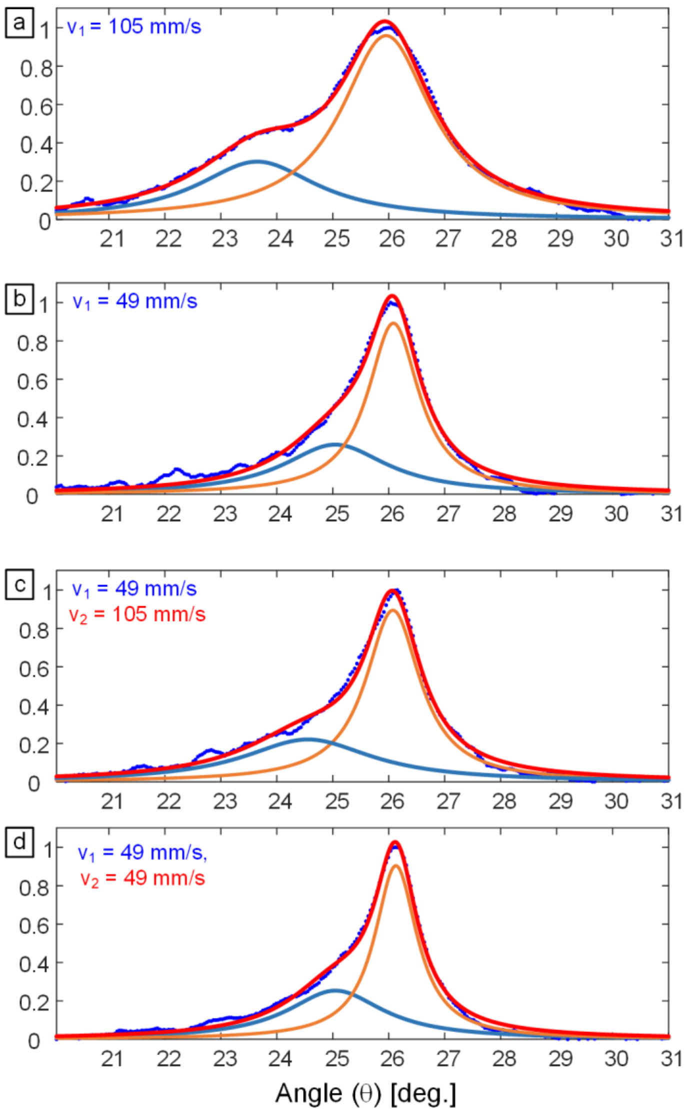

Figure S8. Plot of deconvolution of (002) peak in XRD profiles of LINC lines lased using conditions P = 12.5W, z = 9 mm and (a) v_1 = 105 mm/s, (b) v_1 = 49 mm/s, (c) v_1 = 49 mm/s, v_2 =105 mm/s and (d) v_1 = 105 mm/s and v_2 =49 mm/s.

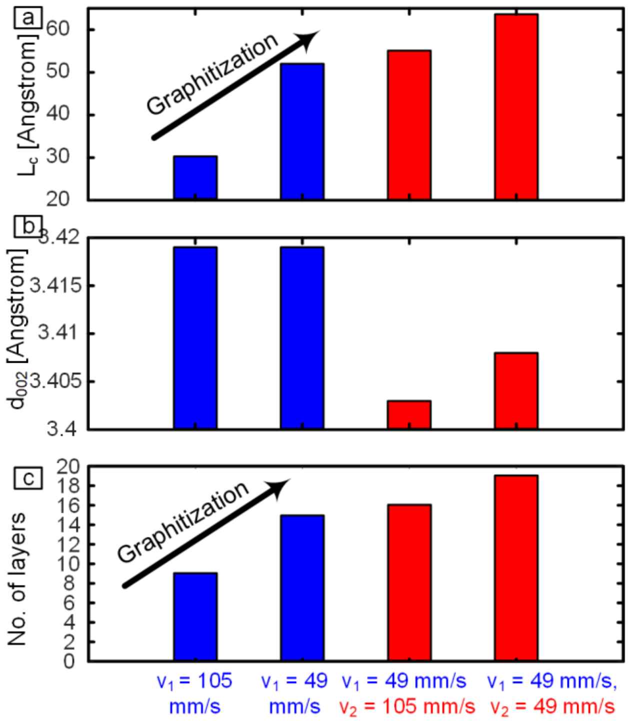

Figure S9. Bar plots demonstrating the change in the (a) crystallite size, (b) spacing and (c) no. of layers of graphitic domain of different LINC lines generated at different laser conditions with P = 12.5W and z = 9 mm.

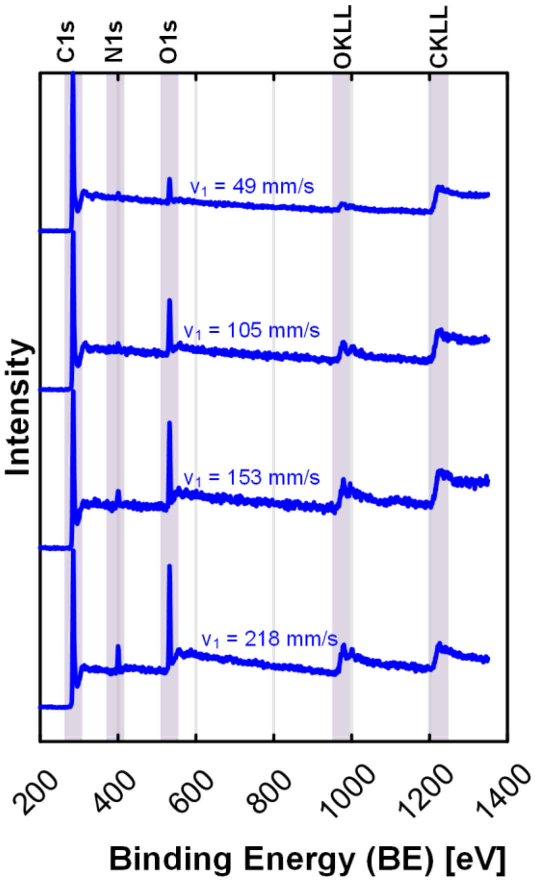

Figure S10. XPS survey scans of LINC lines created using laser condition P = 12.5W, z = 9 mm and different lasing speeds v_1.

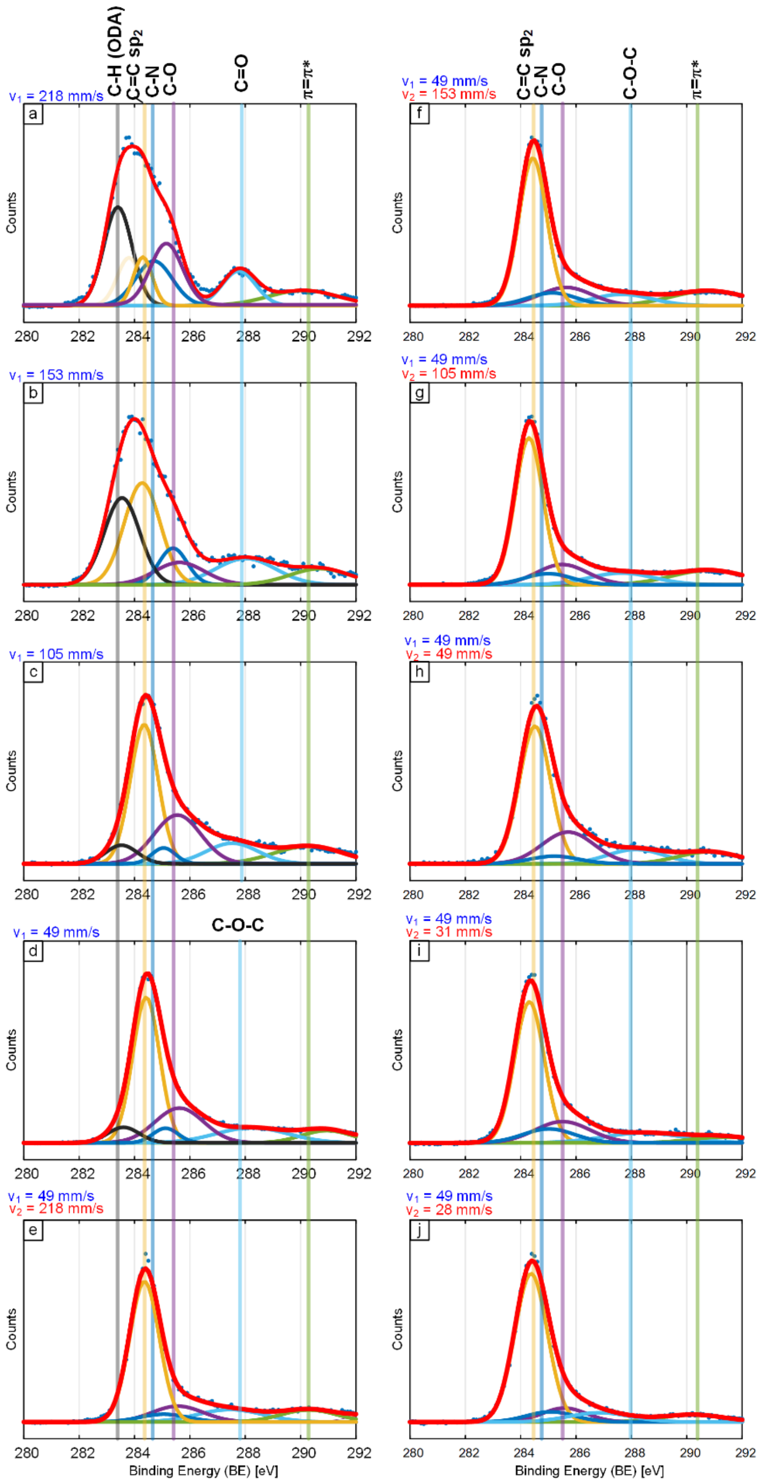

Figure S11. C1s core scans of LINC lines lased using laser conditions P = 12.5 W and different lasing and relasing speeds.

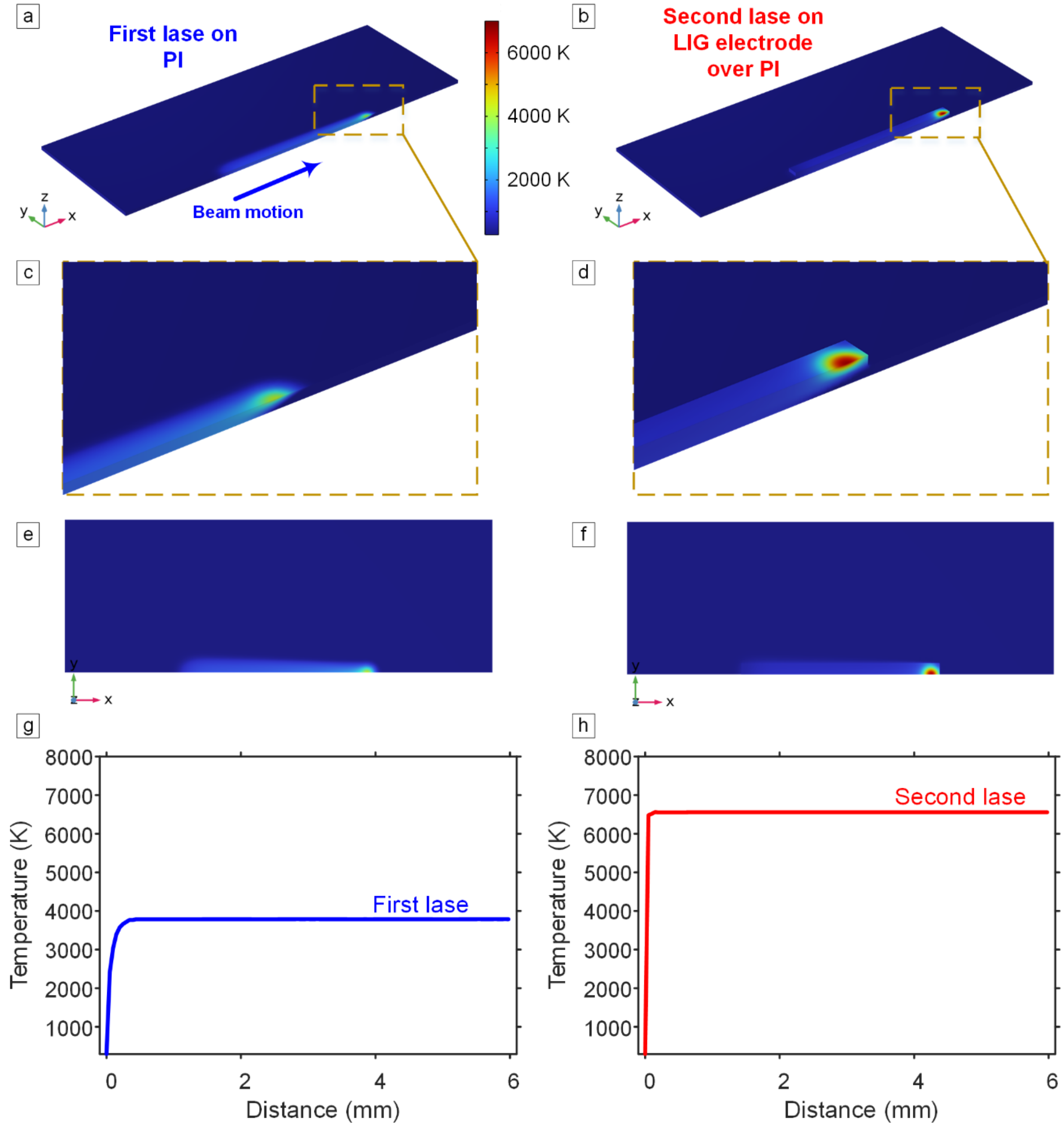

Figure S12. Finite element model for the cases where the laser is irradiated on (a) the PI surface during the first-lase pass, showing the spatial temperature distribution in isometric view after the laser beam has traveled 6 mm, and (b) the LIG electrode on PI during the second-lase pass, showing the temperature distribution in isometric view after the laser beam has traveled 6 mm over the LIG electrode, showing exploded view for (c) First-lase and (d) Second-lase. (e) Top-view temperature distribution for the first-lase case of the PI surface and (f) top-view temperature distribution for the second-lase case of the LIG electrode on PI. (g) Temperature evolution along the beam center as it travels 6 mm for the first-lase case and (h) temperature evolution along the beam center as it travels 6 mm for the second-lase case.

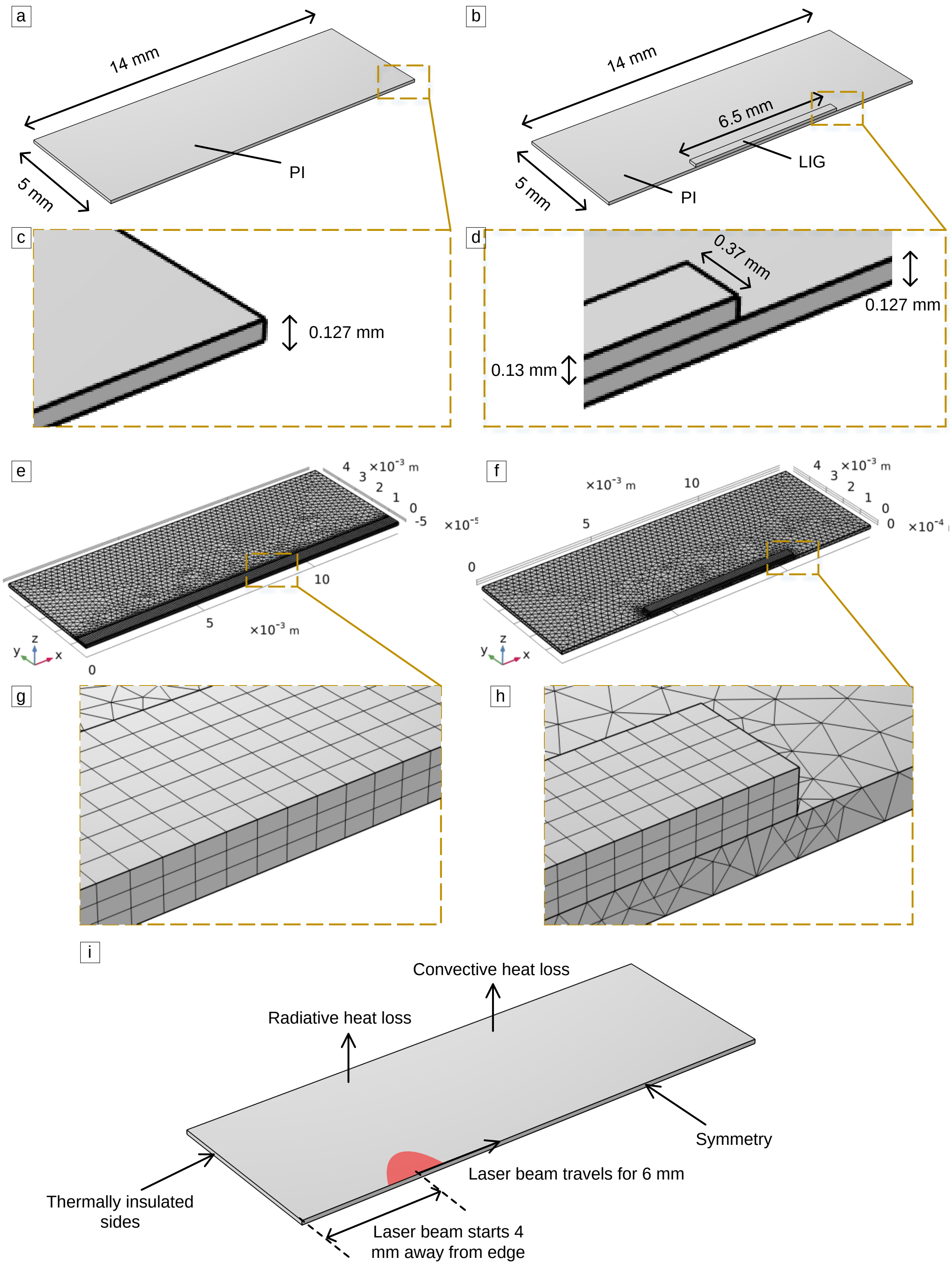
a
14 mm
5 mm
PI
b
14 mm
6.5 mm
LIG
5 mm
PI
c
0.127 mm
d
0.37 mm
0.127 mm
0.13 mm
e
f
g
h
i
Convective heat loss
Radiative heat loss
Symmetry
Laser beam travels for 6 mm
Thermally insulated sides
Laser beam starts 4 mm away from edge

Figure S13. Geometry for the (a) first-lase and (b) second-lase cases, with corresponding insets in (c) and (d) showing the dimensions of the PI substrate and the LIG-on-PI region. (e) Mesh with locally refined elements along the laser beam path for the first-lase case on PI and (f) mesh with locally refined elements along the laser beam path for the second-lase case on LIG over PI. (g) Exploded view of the mesh for the single-lase geometry and (h) exploded view of the mesh for the second-lase geometry. (i) Applied boundary conditions for the single-lase case, which are identical for the double-lase case except for the modified geometry including the LIG electrode line over which the laser beam travels.

Laser heating during the first-lase and second-lase steps was modeled by applying a moving Gaussian surface heat flux on the top surface of the domain. Polymer laser processing is inherently complex, involving coupled laser–material interactions, phase transformations, and chemical reactions occurring over multiple time scales. The present model is therefore simplified but is designed to capture the key temperature evolution driven by laser heating and to provide insight into the differences between the two passes.

Two configurations were simulated: (i) a first-lase case, in which the laser scans over a PI substrate, and (ii) a second-lase case, in which the laser scans over a pre-formed LIG line on PI. The corresponding geometries and boundary conditions are summarized in SI Fig. S18. The dimensions of LIG electrode is estimated from the cross-sectional SEM. All simulations were carried out using the Transient Heat Transfer in Solids module in COMSOL Multiphysics. Exploiting symmetry, only half of the full geometry was modeled, as illustrated in SI Fig. S13, with a symmetry plane along the centerline of the electrode.

Convective heat transfer boundary conditions were imposed on the top and bottom surfaces, with heat transfer coefficients h1=17.9 $Wm^{-2}K^{-1}$ and h2=9.0 $Wm^{-2}K^{-1}$ for the upper and lower surfaces, respectively[1]. On the top surface, thermal radiation to the surroundings was also included using a surface-to-ambient radiation boundary condition with ambient temperature 293 K and emissivity 0.9. The moving Gaussian surface heat flux was implemented using the standard Gaussian expression of the form

$$Q(x,y) = \frac{2P}{\pi\omega_0^2} exp\left(-2\left(\left(\frac{x-vt}{\omega_0}\right)^2 + \left(\frac{y-y_0}{\omega_0}\right)^2\right)\right)$$

with the beam center position of beam spot size ω_0 updated in time t according to the prescribed scan speed v. The laser beam parameters were P = 12.5 W, speed = 49 mm/s, z = 9 mm. At this defocus the beam spot size is 500 microns.

Material properties were taken to be temperature dependent in order to partially account for the changes in thermal behavior associated with carbonization and graphitization during processing.

Temperature-dependent properties were estimated based on literature values for PI-derived carbon[2–5]. The PI layer was modeled using temperature-dependent thermal conductivity (K), specific heat capacity (C_p), and density (ρ):

$$K\_PI = \begin{cases} 0.213 + 3.416 \times 10^{-5} \ \left[\frac{W}{m.K}\right], 200\ K < T < 729\ K \\ -1.314 + 2.13 \times 10^{-3} \ \left[\frac{W}{m.K}\right], 729\ K < T < 1500\ K \\ 1.88 \ \left[\frac{W}{m.K}\right], T > 1500\ K \end{cases}$$

$$C_p\_PI = \begin{cases} 1000(0.96 + 1.39(\frac{T-300}{400})\_0.42(\frac{T-300}{400})^2) \ \left[\frac{J}{kg.K}\right], 200\ K < T < 915\ K \\ 2080 \ \left[\frac{J}{kg.K}\right], 915\ K < T < 1800\ K \\ -0.000143 \times T^2 + 0.734 \times T + 1220 \ \left[\frac{J}{kg.K}\right], T > 1800\ K \end{cases}$$

ρ_PI = 1420 [kg/m^3]

The LIG layer was assigned the following material properties[6,7]:

K_PI = 0.85 $\left[\frac{W}{m.K}\right]$

C_p_PI = 2024 $\left[\frac{J}{kg.K}\right]$

ρ_PI = 40 [kg/m^3]

The finite element mesh is shown in SI Fig. S13. Along the laser scan path, the element size was refined to approximately 7.5×10^{-5} m in both the first-lase and second-lase simulations, ensuring comparable spatial resolution of the temperature gradients in the two cases.

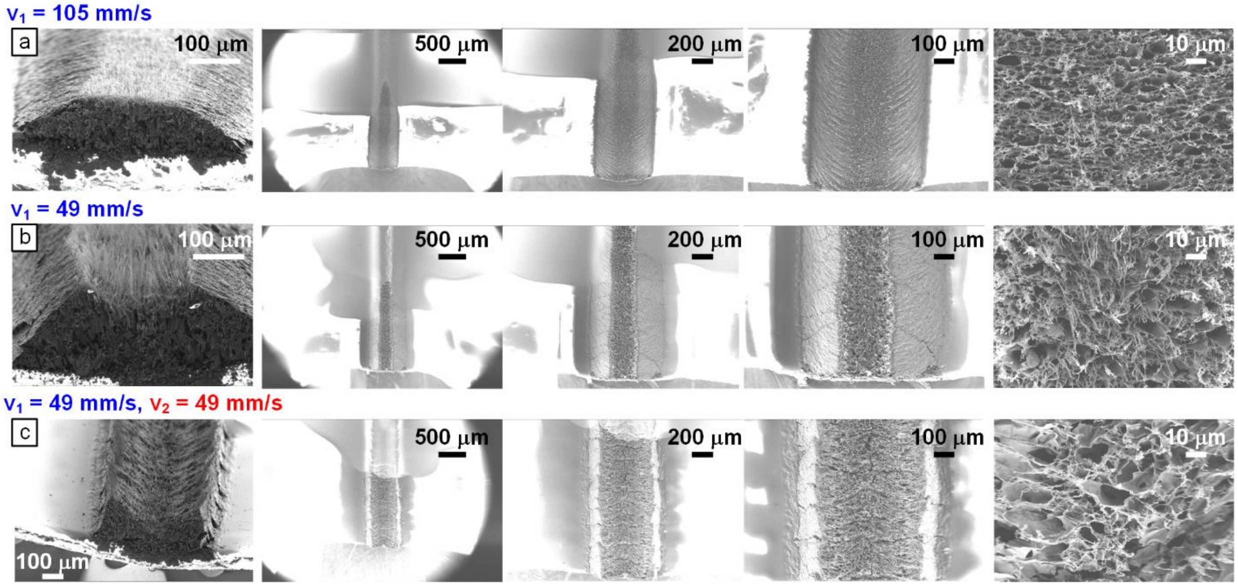

Figure S14. SEM images of LINC neural probes electrodes after packaging for different laser conditions P = 12.5W, z = 9 mm and (a) v_1 = 105 mm/s, (b) v_1 = 49 mm/s and (c) v_1 = 49 mm/s, v_2 = 49 mm/s. showing illustrating the insulation and the surface morphology.

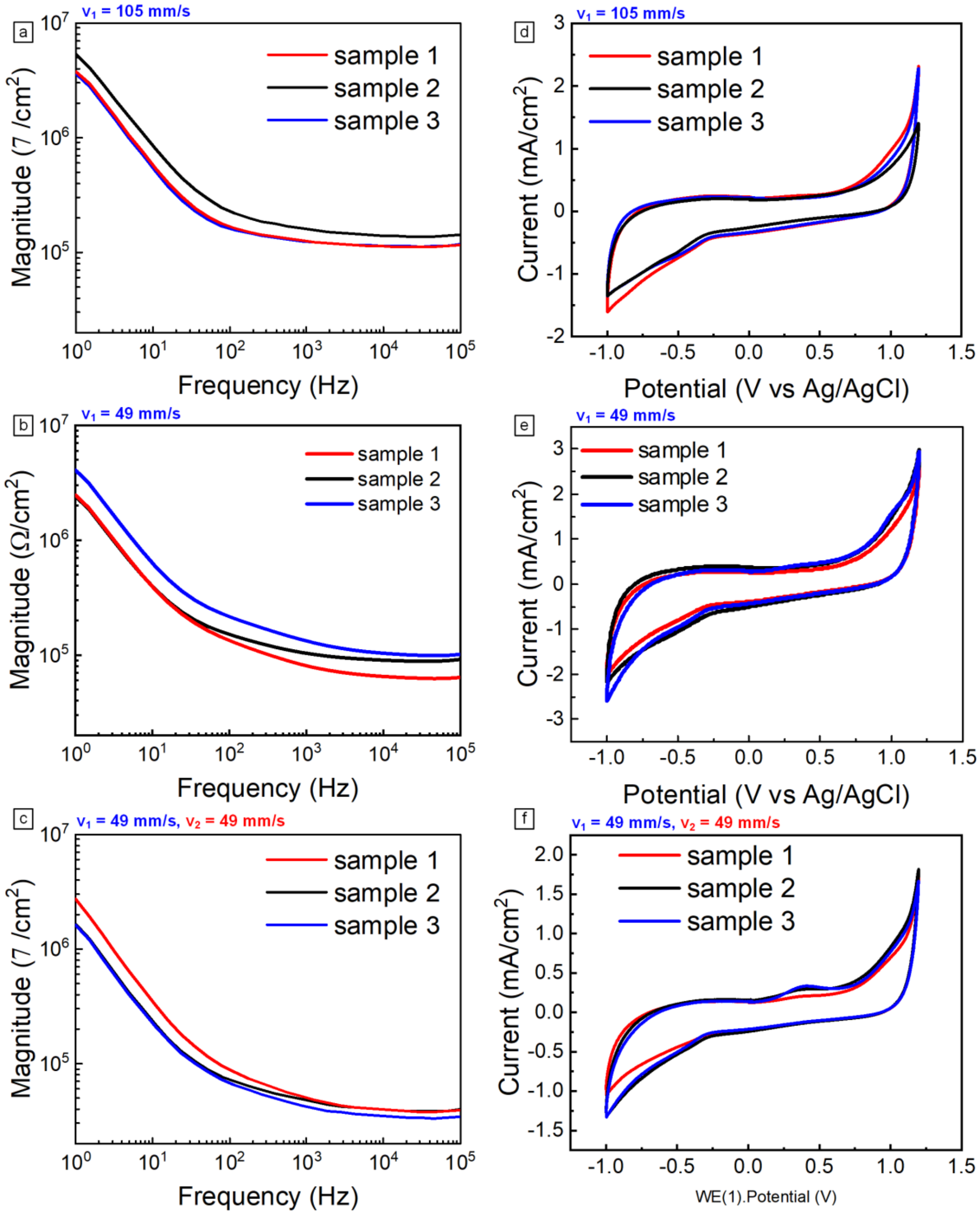

Figure S15. (a-c) Electrochemical impedance spectroscopy plots generated using LINC electrodes at different laser conditions. (d-f) CV plots generated using LINC electrodes at different laser conditions with three sample repeats for each condition.

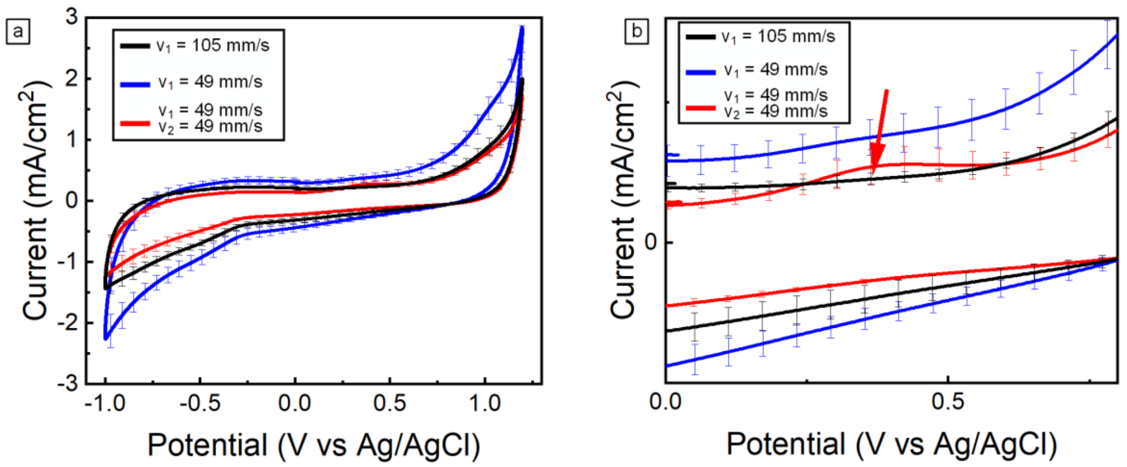

Figure S16. (a) Plot representing area normalized averaged CV results for electrodes created using different laser conditions. (b) Zoomed in CV plot illustrating the more pronounced peak associated with relasing.

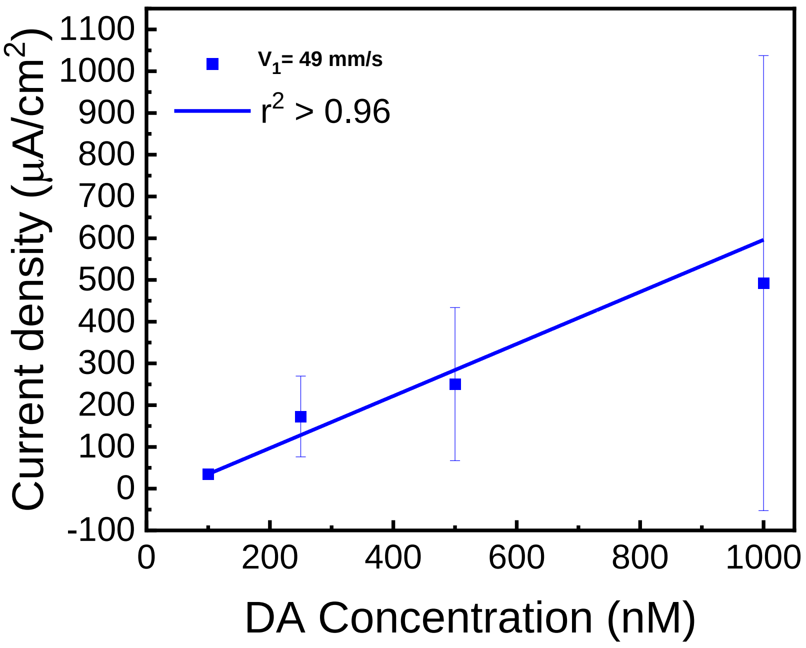

Figure S17. SWV sensitivity of tonic dopamine (DA) concentrations**.** DA sensitivity for LINC electrodes fabricated at P = 12.5W, z = 9 mm and v1 = 49 mm/s lasing conditions. Calibration curves of dopamine in 1x PBS performed show that average DA sensitivities (peak current vs DA concentration) are not linearly correlated and the sample present high variability, corresponding to a very high standard deviation.

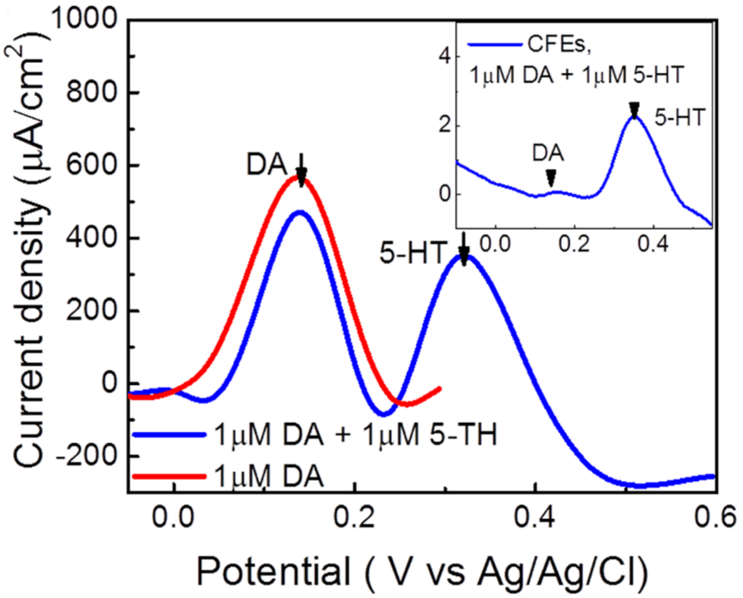

Figure S18. Baseline-subtracted SWV of 1 μM DA (red) and 1 μM DA + 1 μM 5-H mixture from LINC (blue) Inset: baseline subtracted peaks 1 μM DA + 1 μM 5-H mixture from carbon fiber microelectrode (CFE).

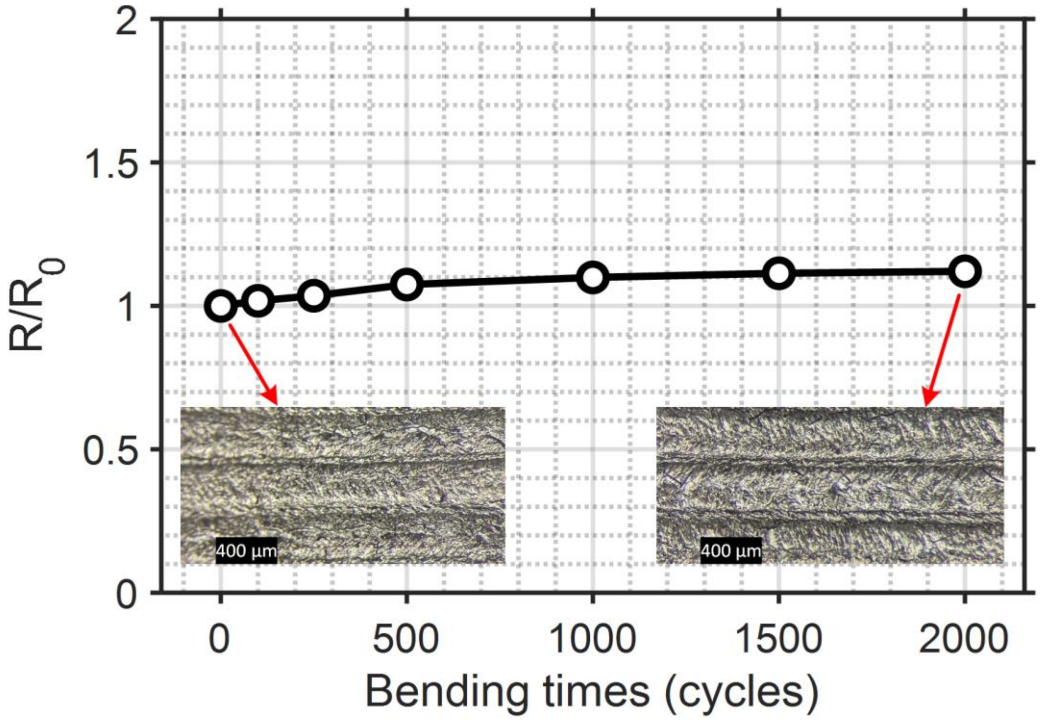

Figure S19. Normalized resistance (R/R_0) as a function of bending cycles. The plot shows the variation in electrical resistance of the sample relative to its initial value (R_0) during repeated bending to a diameter of 8 mm. The fully covered LIG sample exhibits minimal change in resistance (< 11%) even after 2000 cycles. The insets display optical microscopy images of the sample before and after the bending test. (P = 12.5 W, speed = 111 mm/s, z = 6 mm, and raster gap = 355 µm)

Table S1: Summary of XRD deconvolution and derived crystallite parameters.

v_1 [mm/s]	105	49	49	49
v_2 [mm/s]			105	49
2θ [deg.]	26.034	26.034	26.154	26.114
FWHM [deg.]	2.653	1.550	1.463	1.266
L_c [Å]	30.381	51.993	55.103	63.682
d_{002} [Å]	3.419	3.419	3.403	3.408

Table S2: Summary of C1s peak deconvolution

v1 = 218 mm/s			
Peak Name	Position (eV)	Area	FWHM (eV)
C-H (ODA)	283.8	0.75	1.2
C-H (PMDA)	284.2	0.304	1
C=C sp2	284.7	0.246	0.8
C-N	285.1	0.441	1.56
C-O	285.55	0.528	1.36
C=O	288.17	0.311	1.42
Pi-Pi*	290.5	0.285	2.95
v1 = 153 mm/s			
Peak Name	Position (eV)	Area	FWHM (eV)
C-H (ODA)	283.972	0.829	1.506
C=C sp2	284.7	0.987	1.531
C-N	285.815	0.278	1.2
C-O	286.051	0.297	2.113
C=O	288.434	0.422	2.55
Pi-Pi*	291.124	0.259	2.554
v1 = 105 mm/s			
Peak Name	Position (eV)	Area	FWHM (eV)
C-H (ODA)	283.88	0.159	1.346
C=C sp2	284.7	1.05	1.191
C-N	285.4	0.1	0.995
C-O	285.9	0.615	1.992
C=O	287.88	0.287	2.2
Pi-Pi*	290.65	0.33	2.95
v1 = 49 mm/s			
Peak Name	Position (eV)	Area	FWHM (eV)
C-H (ODA)	283.88	0.132	1.342
C=C sp2	284.7	1.057	1.148
C-N	285.401	0.1	1.074
C-O	285.901	0.454	2.059
C-O-C	288.524	0.292	3.042
Pi-Pi*	291.233	0.166	2.132
v1 = 49 mm/s, v2 = 218 mm/s			
Peak Name	Position (eV)	Area	FWHM (eV)
C=C sp2	284.7	1.114	1.252
C-N	285.4	0.1	2.029
C-O	285.9	0.218	2.166
C-O-C	287.798	0.218	2.778
Pi-Pi*	290.641	0.181	2.373

v1 = 49 mm/s, v2 = 153 mm/s			
Peak Name	Position (eV)	Area	FWHM (eV)
C=C sp2	284.7	1.101	1.178
C-N	285.4	0.166	2.069
C-O	285.9	0.255	2.213
C-O-C	287.925	0.18	2.514
Pi-Pi*	291.06	0.295	3.162
v1 = 49 mm/s, v2 = 105 mm/s			
Peak Name	Position (eV)	Area	FWHM (eV)
C=C sp2	284.7	1.103	1.187
C-N	285.4	0.141	2.01
C-O	285.9	0.275	2.152
C-O-C	288.048	0.201	2.73
Pi-Pi*	291.2	0.286	3.134
v1 = 49 mm/s, v2 = 49 mm/s			
Peak Name	Position (eV)	Area	FWHM (eV)
C=C sp2	284.7	1.154	1.325
C-N	285.4	0.1	2.076
C-O	285.9	0.445	2.215
C-O-C	288.341	0.217	2.398
Pi-Pi*	290.985	0.197	2.6
v1 = 49 mm/s, v2 = 31 mm/s			
Peak Name	Position (eV)	Area	FWHM (eV)
C=C sp2	284.7	1.123	1.257
C-N	285.4	0.193	2.184
C-O	285.9	0.317	2.346
C-O-C	288.816	0.211	3.361
Pi-Pi*	291.723	0.1	2.687
v1 = 49 mm/s, v2 = 29 mm/s			
Peak Name	Position (eV)	Area	FWHM (eV)
C=C sp2	284.7	1.298	1.382
C-N	285.4	0.131	2.038
C-O	285.9	0.166	1.886
C-O-C	287.179	0.153	2.616
Pi-Pi*	290.551	0.123	2.649

Table S3: Average Impedance and CSC values for the electrodes

v_1 [mm/s]	v_2 [mm/s]	Impedance @ 1kHz	Impedance @ 100 kHz	CSC
105		102.14±24.90 kW/cm2	85.57±19.41 kW/cm2	2.68±0.29 mC/cm2
49		134.00±19.86 kW/cm2	125.88±14.44 kW/cm2	1.84±0.24 mC/cm2
49	49	45.10±3.95 kW/cm2	37.88±2.91 kW/cm2	1.46±0.14 mC/cm2

References

(1) Ruan, X.; Wang, R.; Luo, J.; Yao, Y.; Liu, T. Experimental and Modeling Study of CO2 Laser Writing Induced Polyimide Carbonization Process. *Mater. Des.* **2018**, *160*, 1168–1177. https://doi.org/10.1016/j.matdes.2018.10.050.

(2) Taylor, R. E.; Groot, H. Thermophysical Properties of POCO Graphite. *High Temp. - High Press.* **1980**, *12* (2), 147–160.

(3) Küper, S.; Brannon, J.; Brannon, K. Threshold Behavior in Polyimide Photoablation: Single-Shot Rate Measurements and Surface-Temperature Modeling. *Appl. Phys. A* **1993**, *56* (1), 43–50. https://doi.org/10.1007/BF00351902.

(4) Kotel'nikov, G. V; Sidorovich, A. V. Microcalorimeter Type DSM-2M for Use in the Study of Polymers. *Polym. Sci. U.S.S.R.* **1983**, *25* (12), 3053–3059. https://doi.org/https://doi.org/10.1016/0032-3950(83)90060-6.

(5) Venkatachalam, S.; Depriester, M.; Sahraoui, A. H.; Capoen, B.; Ammar, M. R.; Hourlier, D. Thermal Conductivity of Kapton-Derived Carbon. *Carbon N. Y.* **2017**, *114*, 134–140. https://doi.org/https://doi.org/10.1016/j.carbon.2016.11.072.

(6) Kincal, C.; Solak, N. Controlling Thermoelectric Properties of Laser-Induced Graphene on Polyimide. *Nanomaterials*. 2024, p 879. https://doi.org/10.3390/nano14100879.

(7) Yang, D.; Nam, H. K.; Lee, Y.; Kwon, S.; Lee, J.; Yoon, H.; Kim, Y.-J. Laser-Induced Graphene Smart Textiles for Future Space Suits and Telescopes. *Adv. Funct. Mater.* **2025**, *35* (1), 2411257. https://doi.org/https://doi.org/10.1002/adfm.202411257.
